# Ultra-long-range interactions between active regulatory elements

**DOI:** 10.1101/gr.277567.122

**Published:** 2023-08

**Authors:** Elias T. Friman, Ilya M. Flyamer, Davide Marenduzzo, Shelagh Boyle, Wendy A. Bickmore

**Affiliations:** 1 MRC Human Genetics Unit, Institute of Genetics and Cancer, University of Edinburgh, Edinburgh EH4 2XU, United Kingdom;; 2 School of Physics and Astronomy, University of Edinburgh, Edinburgh EH9 3FD, United Kingdom

## Abstract

Contacts between enhancers and promoters are thought to relate to their ability to activate transcription. Investigating factors that contribute to such chromatin interactions is therefore important for understanding gene regulation. Here, we have determined contact frequencies between millions of pairs of *cis*-regulatory elements from chromosome conformation capture data sets and analyzed a collection of hundreds of DNA-binding factors for binding at regions of enriched contacts. This analysis revealed enriched contacts at sites bound by many factors associated with active transcription. We show that active regulatory elements, independent of cohesin and polycomb, interact with each other across distances of tens of megabases in vertebrate and invertebrate genomes and that interactions correlate and change with activity. However, these ultra-long-range interactions are not dependent on RNA polymerase II transcription or individual transcription cofactors. Using simulations, we show that a model of chromatin and multivalent binding factors can give rise to long-range interactions via bridging-induced clustering. We propose that long-range interactions between *cis*-regulatory elements are driven by at least three distinct processes: cohesin-mediated loop extrusion, polycomb contacts, and clustering of active regions.

The large genomes of most animal species derive their regulatory potential from noncoding *cis*-regulatory elements (CREs), in particular the activation of genes by enhancers. The spatial relationship between CREs and transcription is a topic of active debate (see, e.g., Benabdallah et al. 2019; Mir et al. 2019; Xiao et al. 2021; Karr et al. 2022; Zuin et al. 2022), and thinking in this area has been strongly influenced by genome-wide chromosome conformation capture methods such as Hi-C (Lieberman-Aiden et al. 2009) and Micro-C (Hsieh et al. 2015, 2020). Contact frequency, which is the measure of how often two regions are in proximity for cross-linking and ligation, strongly scales negatively with the genomic distance between two regions, meaning that, on average, regions interact less the more intervening chromatin is between them (Lieberman-Aiden et al. 2009).

CREs controlling developmentally regulated genes are typically contained within a single topologically associating domain (TAD), formed by the highly dynamic process of cohesin-mediated loop extrusion (Symmons et al. 2014; Gabriele et al. 2022). Regions within a TAD interact more frequently with each other than with regions at similar genomic distances outside the TAD. This is because cohesin can extrude chromatin until it reaches barrier elements, most notably CTCF sites, which delineate TAD boundaries (Fudenberg et al. 2016; Nora et al. 2017). Furthermore, focal contacts and “stripes” are seen between pairs of CTCF sites in the presence of loop extrusion (Rao et al. 2014, 2017). Both TADs and cohesin-driven focal contacts are limited to regions separated by up to 1–2 Mb (Dixon et al. 2012; Rao et al. 2017). Much less understood are mechanisms that lead to enriched interactions between regions over larger genomic distances, which may be independent of cohesin.

One process bringing distal regions together is compartment interactions with their own chromatin type, within and between chromosomes. Initial analysis revealed A and B domains, corresponding roughly to active and inactive chromatin, that are several hundred kilobases to a few megabases in size (Lieberman-Aiden et al. 2009). More fine-scale mapping revealed “subcompartments” within these larger domains (Rao et al. 2014; Spracklin et al. 2023), with extremely high sequencing depths revealing compartments of sizes down to a few kilobases (Goel et al. 2023; Harris et al. 2023). What drives compartment interactions is not fully understood, although perturbation of transcription in *Drosophila melanogaster* or DNA methylation in human cell lines can affect interactions between active or inactive compartments, respectively (Rowley et al. 2017; Spracklin et al. 2023). Compartment interactions may be driven at least in part by affinity between molecules differentially present in compartments, such as histone marks, and might also involve phase separation (Nuebler et al. 2018; Falk et al. 2019; Nichols and Corces 2021).

In repressed chromatin, enriched focal interactions are detected between polycomb-bound regions over a very wide range of distances, up to several tens of megabases (Joshi et al. 2015; Bonev et al. 2017; Boyle et al. 2020; Loubiere et al. 2020; Rhodes et al. 2020). These interactions are cohesin independent but are dependent on components of the polycomb repressive complexes. The functional significance of these focal interactions in polycomb-mediated repression is unclear (Ogiyama et al. 2018; Dimitrova et al. 2022).

There is also evidence for associations between active genomic regions over very large genomic distances. TADs containing superenhancers and active genes are spatially closer to other highly active TADs than to those with low activity (Beagrie et al. 2017), and active regions tend to colocalize with nuclear speckles or with RNA polymerase II (Pol II) and other active genes in transcriptional hubs (Osborne et al. 2004; Schoenfelder et al. 2010; Chen et al. 2018; Quinodoz et al. 2018). In mouse embryonic stem cells (mESCs) and differentiated cell types, active transcription start sites (TSSs) and transcription factor (TF) binding sites have been shown to interact with their own type across 2–10 Mb (Bonev et al. 2017). The function of, and processes driving, these long-range interactions and whether they also occur in other cell types and species are not known.

In this study, we use a large collection of genome-wide binding and contact frequency data as well as computational modeling with the aim of characterizing and classifying different processes driving chromatin interactions between CREs at different scales.

## Results

### Screening for factors bound at sites of enriched CRE–CRE interactions

**Figure 1.**
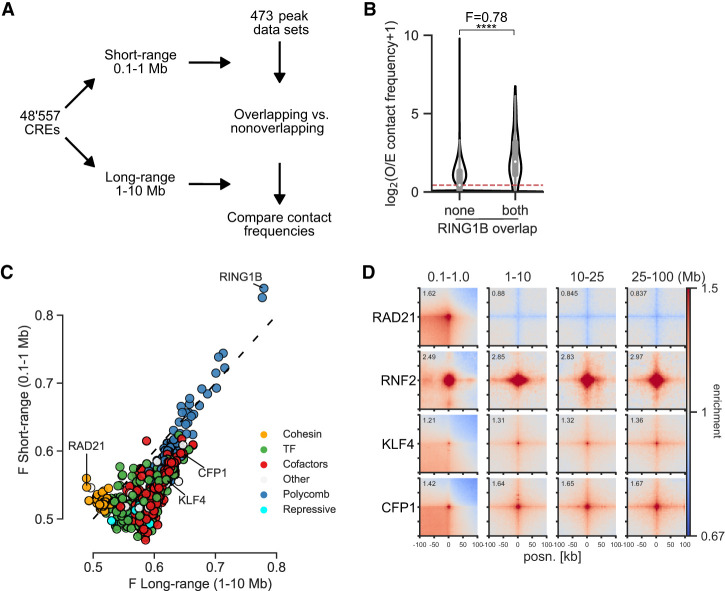
Computational screening for factors associated with enriched interactions between CREs. (*A*) Schematic of the computational pipeline. CRE pairs are split into short range (0.1–1 Mb between pairs) and long range (1–10 Mb between pairs). These regions are overlapped with peaks from different data sets and contact frequencies at 10-kb resolution compared between pairs overlapped by the factor and between pairs not overlapped by the factor to calculate enrichment values. (*B*) Example of the analysis for a RING1B ChIP-seq data set. Observed over expected (O/E) contact frequencies for CREs at long range were split into overlapping RING1B peaks on neither or both sides, and Mann–Whitney *F* and adjusted *P*-values were calculated: (****) *P* = 3.8 × 10^−184^. (*C*) Effect sizes for factors with significantly enriched chromatin interactions compared with unbound CREs in mESCs. The *x-* and *y*-axes show enrichment at short range and long range. Colors represent the group to which the factor belongs. (*D*) Pileup analysis for 5000 regions for each of the indicated hits from *C* for interaction pairs at different distances of genomic separation in Micro-C data from mESCs. For CFP1 and KLF4, peaks overlapping RING1B binding sites were excluded.

To quantify interactions between CREs, we merged DNase-accessible ENCODE (Luo et al. 2020) CREs from mESCs within 5 kb of each other and generated observed over expected (O/E) contact frequencies for each CRE pair from Hi-C and Micro-C data at both short range (0.1–1 Mb) and long range (1–10 Mb) (Fig. 1A). The 10-Mb cut-off was chosen to limit the number of combinations and to avoid sparse data at larger distances. We determined the overlap of CREs with binding sites (peaks) in the Cistrome DB (Mei et al. 2017) and ReMap2022 (Hammal et al. 2022) databases. For each peak data set representing one ChIP-seq experiment, we divided CREs into overlapping and nonoverlapping. We then compared the contact frequencies of CRE pairs overlapping the factor on both sides, or not at all, by calculating the Mann–Whitney *U* adjusted *P*-value and effect size (F = U/(n_1_ × n_2_)), where F > 0.5 means increased contact frequencies at bound compared with unbound (Fig. 1B). We annotated the factors into six classes: cohesin-associated, polycomb-associated, transcription cofactors, TFs, repressive factors, and other. We use transcription cofactors broadly here to mean chromatin and transcription-associated proteins that are not known TFs or repressors.

We tested our approach by measuring changes in enrichment in the six different classes in Micro-C and Hi-C data sets from cells in which cohesin-associated factors had been degraded (Hsieh et al. 2022) or *Rnf2* (also known as *Ring1B*) (polycomb) knocked out (Boyle et al. 2020). As expected, RAD21 and CTCF degradation led to a decrease (Supplemental Fig. S1A,B) and WAPL degradation led to an increase (Supplemental Fig. S1C) in enrichment between cohesin-associated binding sites at short range. *Rnf2* knockout (KO) led to an expected decrease in enrichment for polycomb-bound regions at both short and long range (Supplemental Fig. S1D).

After confirming that our approach was able to detect factors associated with enriched chromatin interactions, we performed our analysis on Micro-C data from WT mESCs (Fig. 1C; Supplemental Fig. S2A,B; Supplemental Table S1; Hsieh et al. 2020). Cohesin-associated factors were enriched for interactions at short but not long range, and polycomb-associated factors were similarly enriched at short and long range. Besides these already known mechanisms, binding sites for transcription cofactors and TFs tended to be enriched at both short and long range, but with higher enrichment at long range. We correlated the number of non-polycomb-bound TSSs per CRE bound by each factor and its enrichment. TFs and cofactors had a high correlation between enrichment and TSS overlap, showing that they are separate from polycomb and related to the presence of genes (Supplemental Fig. S2C). This is not a feature specific to Micro-C data or to mESCs as we also saw a similar pattern of enrichment for the different classes in Hi-C data from the GM12878 human lymphoblastoid cell line (Supplemental Fig. S2D–F; Supplemental Table S2; Rao et al. 2014). We used pileup analysis (Flyamer et al. 2020) to confirm some of the top hits. Sites bound by the CpG island (CGI) binding protein CFP1 (also known as CXXC1) or by KLF4, a pluripotency TF active in mESCs, showed enrichment at all distances up to 100 Mb (Fig. 1D). We call this noncohesin, non-polycomb-associated category of interactions between active regions at large distances “ultra-long-range interactions” (ULIs).

ULIs are seen as enriched stripes and central pixels in pileups, meaning that these active elements interact with each other and with the surrounding chromatin more than with other regions at similar distances. Note that although the level of enrichment above expected is similar across distances, this does not represent similar absolute contact frequencies. We considered potential technical artifacts that could lead to the appearance of ULIs. We divided accessible regions into quartiles based on DNase-seq signal, split them into those within 1 kb of a TSS or at least 5 kb from the nearest TSS, and generated pileups (Supplemental Fig. S3A,B). Although ULIs scaled with accessibility, TSSs in lowly accessible regions were much more enriched for ULIs than more accessible sites without TSSs, arguing against this signal simply being a result of increased digestion or cross-linking efficiency in accessible chromatin. We also considered that large protein complexes bound at active genes could lead to increased cross-linking efficiency. However, contact frequencies did not correlate with the number of Cistrome DB and ReMap2022 peaks overlapping CREs, suggesting this is not the case (Supplemental Fig. S3C,D). We also excluded normalization artifacts, as ULIs can be seen in both unbalanced and balanced (iterative convergence and eigenvector decomposition [ICE] normalized) (Imakaev et al. 2012) data, whether balanced on all, or only on *cis*, contacts (Supplemental Fig. S3E). ULIs are also seen regardless of normalizing by expected values, shifted controls, or not at all. We therefore conclude that ULIs represent bona fide enriched contacts between active regulatory elements.

### ULIs between active regions are independent of cohesin and polycomb

**Figure 2.**
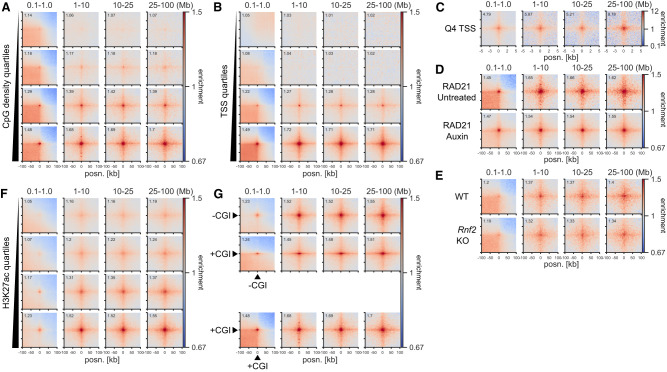
Ultra-long-range interactions (ULIs) between active promoters and distal regulatory elements. Pileup analysis. (*A*) Non-RNF2 overlapping CGIs split by quartiles based on CpG density (2352–3015 peaks per group). (*B*) Non-RNF2 overlapping TSSs (4800–5128 peaks per group) divided into quartiles by expression level, based on 4sU-seq data, in Micro-C data from mESCs. (*C*) The 10 kb surrounding the Q4 TSSs (5128 peaks) at 100-bp resolution in Micro-C data from mESCs. (*D*,*E*) CGI Q4 (2651 peaks) regions in Micro-C data from RAD21-AID mESCs (*D*) and Hi-C data from WT or *Rnf2* KO mESCs (*E*). (*F*) TSS-distal H3K27ac peaks not overlapping RNF2 or CGIs split by quartiles based on H3K27ac ChIP-seq signal (6161–6452 peaks per group) in Micro-C data from mESCs. (*G*) CGI Q4 regions (2651 peaks; +CGI) and Q4 promoter-distal H3K27ac (6268 peaks; −CGI) in Micro-C data from mESCs.

The strong enrichment for CFP1 binding sites in our screen (Fig. 1C; Supplemental Fig. S2B) prompted us to test the relationship between CGIs and ULIs. This showed a correlation between interactions and the density of CpG dinucleotides at CGIs devoid of polycomb (Fig. 2A). Interaction strength at TSSs also scaled strongly with the level of transcription (Fig. 2B). Notably, TSSs in quartile 4 (highly transcribed) showed enrichment with quartiles 3 and 2 but not with quartile 1, meaning that inactive genes do not interact at all (Supplemental Fig. S4A). Non-CGI promoters also interact but much less than CGI promoters, suggesting CGIs are not required but contribute to ULIs at TSSs (Supplemental Fig. S4B). Analysis of Micro-C data at 100-bp resolution within the 10-kb region surrounding TSSs showed that enriched interactions are centered at the TSS (Fig. 2C).

We confirmed that similar to polycomb-dependent contacts but unlike cohesin-driven interactions, interactions between active regions at short distances (<1 Mb) span TAD boundaries (Supplemental Fig. S4C). This indicates that the processes that drive ULIs also operate at shorter distances and are independent of loop extrusion. To formally show that ULIs are not dependent on polycomb and cohesin, we analyzed data from mESCs with *Rnf2* deleted (KO) or RAD21 degraded (AID) for 3 h, which disrupts polycomb- and CTCF-mediated interactions, respectively (Boyle et al. 2020; Hsieh et al. 2022). These interventions did not affect ULIs (Fig. 2D,E) and neither did 3 h of depletion of CTCF or the cohesin unloader WAPL (Supplemental Fig. S4D). These results are in line with many shorter-range enhancer–promoter interactions being largely independent of cohesin and CTCF (Hsieh et al. 2022). However, we did note a strong reduction of interactions after 6 h of RAD21 depletion in an independent mESC data set (Supplemental Fig. S4E; Rhodes et al. 2020) but not in data from *Rad21* KO noncycling mouse thymocytes (Supplemental Fig. S4F; Seitan et al. 2013). Furthermore, in human HAP1 cells (Haarhuis et al. 2017), we did not detect a loss of ULIs upon deletion of the cohesin subunit *MAU2* (also known as *SCC4*), whereas *WAPL* deletion led to slightly decreased interactions (Supplemental Fig. S5A). It is possible that the stiffening of the chromatin fiber that occurs upon prolonged loss of WAPL (Tedeschi et al. 2013) leads to a less dynamic chromatin structure, preventing active regions from interacting. Overall, of the cohesin subunit perturbations examined, only 6 h of RAD21 degradation in mESCs led to a large reduction of ULIs, whereas shorter (3-h) degradation or complete KO in HAP1 and thymocytes did not. This may be because of the indirect effects of long-term RAD21 depletion in mESCs including, but not limited to, the cell cycle. We cannot exclude that cohesin has an impact in certain contexts, but the above results strongly suggest loop extrusion is not required for ULIs.

Analysis of a Hi-C data set from cell cycle–synchronized erythroblasts (Zhang et al. 2019) showed, as expected, that ULIs are lost in mitosis and reappear as early as anaphase/telophase (Supplemental Fig. S5B). This is before the formation of both CTCF loops and compartments (Zhang et al. 2019). Analysis of cell cycle–phased merged single-cell Hi-C data (Nagano et al. 2017) shows that ULIs persist throughout the G_1_, S, and G_2_ phases (Supplemental Fig. S5C).

Promoter-distal H3K27ac-marked CREs, namely, putative enhancers, which do not overlap with CGIs, also show enriched ULIs that scale with the level of H3K27ac enrichment (Fig. 2F). ULIs also occurred between these distal H3K27ac regions and CGIs, as well as expressed genes (Fig. 2G; Supplemental Fig. S5D). Taken together, these results show that ULIs scale with the activity of regulatory elements, are independent of polycomb and cohesin, are present in cycling and noncycling cells, are reformed quickly after mitosis, and persist throughout the cell cycle.

### Rare interactions between distal CREs

**Figure 3.**
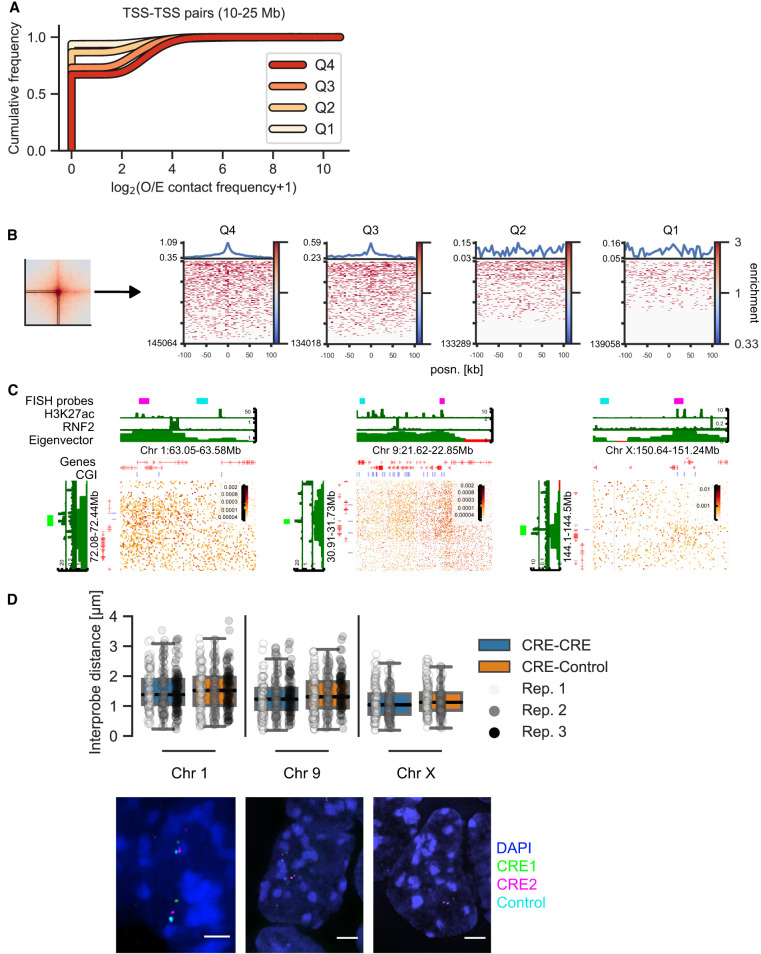
Distribution of contact frequencies and DNA FISH. (*A*) Empirical cumulative distribution frequency of O/E contact frequency values in the central 5-kb pixel between TSSs in different expression quartiles separated by 10–25 Mb. (*B*, *left*) Depiction of the “corner stripe” from a pileup. (*Right*) Heatmaps of O/E contact frequency values in individual corner stripes for TSS–TSS quartile interaction pairs at 10- to 25-Mb distance. The line plot on *top* represents the mean signal. (*C*) Hi-C matrices for regions on Chromosomes 1, 9, and X used in DNA FISH experiments. CGI marks CGIs without RNF2. (*D*, *top*) Interprobe distances measured by DNA FISH between distal CREs on Chromosomes 1, 9, and X or a CRE and an equidistant control region in the same A compartment. N = 289 (Chr 1), 292 (Chr 9), and 208 (Chr X) measurements each for CRE–Control and CRE–CRE. Boxplot lines denote maximum (excluding outliers), interquartile range (*upper* and *lower* bound of box), median (*center* of box), and minimum values. (*Bottom*) Example images of DNA FISH. Blue indicates DAPI; green, CRE1 probe; magenta, CRE2 probe; and cyan, control probe (adjacent to CRE2 in the genome). Scale bar, 2 μm.

Enrichment in the pileups represents averages of many pairs of regions. High average enrichment could come from a few highly interacting pairs or from a tendency of many or all pairs to interact. To test this, we assessed the distribution of contact frequency values using two approaches, focusing on TSS quartile pairs at 10- to 25-Mb distance. First, we quantified the contact frequency in the central 5-kb bin containing the TSS. Higher expression quartiles had higher contact frequencies, although most values were zero in all quartiles, reflecting the sparsity of data at these large distances (Fig. 3A). We also looked at the signal of the individual “corner stripes” in the pileups (Fig. 3B). This revealed a higher number of nonzero values across the stripe with increased expression level (Q3 and Q4). We did not find evidence of a few particularly highly interacting pairs dominating the pileups, showing rather that the observed enrichments are a property of increased interaction frequencies between many pairs.

The analyses above show that ULIs represent higher average contact frequencies between active CREs compared to the surrounding chromatin. Contact frequencies are related to, but are not a direct measure of, spatial distances (Fudenberg and Imakaev 2017; Finn et al. 2019). Nevertheless, we expected that active CREs separated from each other by large genomic distances would be closer together in the nucleus on average than with regions at similar distances that are not active CREs. To test this, we performed DNA FISH on mESC nuclei using probes between pairs of CREs at 9.1, 8.9, and 6.8 Mb of genomic separation or between one CRE and an equidistant control region in the same A compartment (Fig. 3C). CRE–CRE pairs had significantly smaller distances separating them than CRE–Control pairs (linear mixed-effect model, *P* = 0.03); although medians were lower for all individual CRE–CRE pairs, only one of these pairs (Chr X) was statistically significant (Fig. 3D; Supplemental Fig. S5E). This indicates that although the average enrichment between distal CREs is high in pileups, this only translates to marginally smaller distances between individual distal CREs in nuclei (median distance in all cases was >1 micron). We only detected two instances of colocalization (<200 nm distance; both in CRE–CRE pairs) between 3156 total measured distances, showing that the enriched contacts seen between many regions in Hi-C/Micro-C are exceedingly rare between individual pairs (see Discussion).

### ULIs occur primarily in *cis* and in the A compartment

Because ULIs are enriched at such large genomic distances along a chromosome, we tested if they are also enriched in *trans* between chromosomes. Indeed, high CpG-density regions devoid of polycomb are enriched in *trans* interactions (Supplemental Fig. S6A), but although the level of enrichment compared with that expected is comparable to that within chromosomes, the absolute *trans* contact frequencies are very low. We also considered that interactions might occur between homologs and tested this using Hi-C data from hybrid mESCs with phased SNPs (Han et al. 2020). This showed that interactions occur primarily within chromosomes and not between homologs (Supplemental Fig. S6B).

Active regions in the A compartment at distances of several tens of megabases, such as those we look at here, will be separated by multiple intervening A and B compartments. We wondered if the stripe seen in the pileups would span the whole intervening chromatin or be constrained to A compartments. We picked regions close to A/B compartment switches and saw that the interaction enrichment was confined to the A compartment; namely, the stripe does not span into the B compartment (Supplemental Fig. S6C).

### Interaction dynamics and DNA methylation

**Figure 4.**
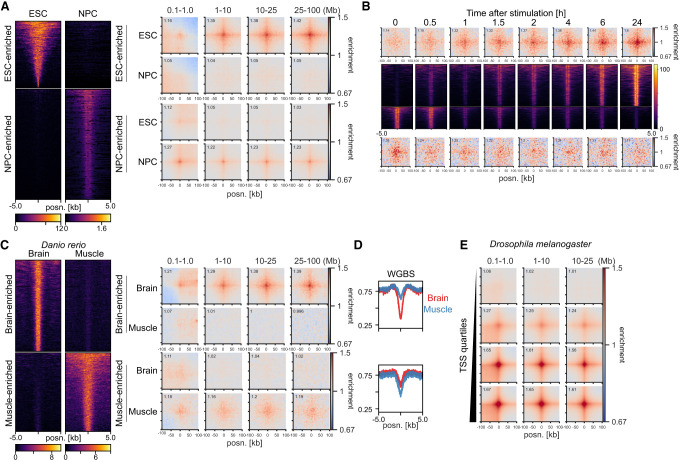
Interaction dynamics in mouse, human, zebrafish, and *Drosophila melanogaster*. (*A*, *left*) H3K27ac signal in mESCs and neural progenitor cells (NPCs) at mESC-enriched (1509 peaks) and NPC-enriched (2491 peaks) regions. (*Right*) Pileup analysis for the differentially enriched H3K27ac peaks in Hi-C from mESCs and NPCs. (*B*, *top* and *bottom*) Pileup analysis for regions with increasing (*top*; 1058 peaks) or decreasing (*bottom*; 553 peaks) H3K27ac across the time course of LPS and IFNG stimulation of THP-1-derived macrophages. (*Middle*) H3K27ac signal in THP1-derived macrophages at regions with increasing (*top*) or decreasing (*bottom*) H3K27ac. (*C*, *left*) H3K27ac signal in zebrafish brain and muscle at brain-enriched (2315 peaks) and muscle-enriched (1994 peaks) regions. (*Right*) Pileup analysis for the differentially enriched H3K27ac peaks in brain and muscle. (*D*) Average whole-genome bisulfite (WGBS) signal in the differentially enriched H3K27ac peaks in brain and muscle. (*E*) Pileup analysis for *D. melanogaster* TSSs (2943 regions per group), divided by expression level based on RNA-seq, in Hi-C data from *D. melanogaster* eye-antennal imaginal discs.

Their relationship to transcriptional activity and H3K27ac suggests that ULIs are dynamic between tissues. To investigate this, we used data from mESCs and differentiated neural progenitor cells (NPCs) (Bonev et al. 2017) and selected H3K27ac peaks enriched in either of the cell states (Fig. 4A). These regions showed correspondingly higher enrichment of ULIs in the cell state with higher H3K27ac. To investigate more rapid dynamics, we examined Hi-C data from human macrophages stimulated with lipopolysaccharide (LPS) and interferon (IFNG) (Fig. 4B; Reed et al. 2022). Regions that gained H3K27ac upon stimulation also gained ULIs, and regions losing H3K27ac, although relatively lowly enriched to begin with, lost ULIs over the time course. Although the small number of regions affected and the time resolution of the experiment precludes delineating which changes come first, it appears that changes in H3K27ac and ULIs accompany one another.

To examine dynamic changes in ULIs between tissues in vivo, we took advantage of data from *Danio rerio* (zebrafish) and selected brain- or muscle-enriched promoter-distal H3K27ac peaks (Yang et al. 2020). These were accompanied by enrichment of ULIs in the respective tissue (Fig. 4C). We also saw a corresponding higher level of DNA methylation in the tissue where the regulatory elements were inactive and no interactions were seen (Fig. 4D). Because of the relationship between CGIs and ULIs (Fig. 2A), we reasoned that the focal demethylation at these regulatory elements may be responsible for the interactions and that gain in DNA methylation would lead to their loss. To test if methylation is required for ULIs, we used Hi-C data from *D. melanogaster*, a species with little DNA methylation and lacking CGIs (Deaton and Bird 2011). We could detect ULIs between expressed TSSs and H3K27ac-positive, non-polycomb-bound (as well as polycomb bound) regions in *D. melanogaster* eye-antennal imaginal discs (Fig. 4E; Supplemental Fig. S6D; Loubiere et al. 2020). This shows that ULIs are not specific to vertebrates and that DNA methylation is not required for their formation.

### ULIs are not directly dependent on transcription

**Figure 5.**
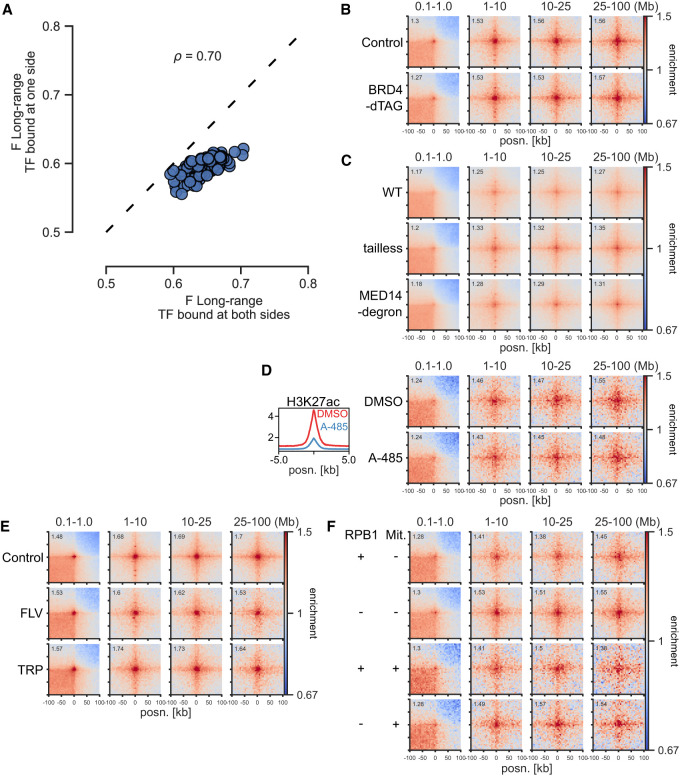
Independence of BRD4, Mediator, EP300 activity, and transcription. (*A*) Effect sizes of TFs for long-range (1- to 10-Mb) contact enrichment compared with TF-unbound CREs in mESCs. The *x-* and *y*-axes show enrichment for TF binding at both sides and only one side, but at least one other TF bound at the other side. (ρ) Spearman's correlation coefficient. (*B*) Pileup analysis between CGI Q4 regions in dTAG-BRD4 mESCs with or without BRD4. (*C*) Pileup analysis between CGI Q4 regions in WT, *Med15*^*−/−*^ *Med16*^*−/−*^ *Med23*^*−/−*^ *Med24*^*−/−*^ *Med25*^*−/−*^ (tailless), and MED14− SMASh degron CH12 cells. (*D*, *left*) Mean H3K27ac signal in CGI Q4 regions in mESCs treated with DMSO or A-485. (*Right*) Pileup analysis between CGI Q4 regions in mESCs treated with DMSO or A-485. (*E*) Pileup analysis between CGI Q4 regions in Micro-C data from WT mESCs or treated with flavopiridol (FLV) or triptolide (TRP) for 45 min. (*F*) Pileup analysis between CGI Q4 regions in RPB1-AID mESCs with or without RPB1. The *top* two rows represent asynchronous cells; the *bottom* two rows represent cells arrested in mitosis (Mit +) and released into G_1_.

In our initial screening, we had seen enrichment for the binding sites of many sequence-specific TFs at ULIs (Fig. 1C; Supplemental Fig. S2A,B). To determine whether homotypic interactions between TFs could drive ULIs, we compared enrichment values for sites where a particular TF is bound at both interacting sides to where that TF is bound at only one side but there is at least one other TF bound at the other side. This is to avoid comparison with regions devoid of binding altogether at one side, namely, inactive regions, which would skew the enrichment. Binding at both sides yielded a stronger enrichment than at one side, but the two were correlated with significant enrichment for every TF also when bound at only one side (Fig. 5A). As an example, we split H3K27ac peaks into those bound by MYC or not in the GM12878 lymphoblastoid cell line and saw that MYC-bound regions interacted with other active regions not bound by MYC (Supplemental Fig. S7A). Although we cannot exclude some contribution, this analysis indicates that homotypic TF–TF interactions are unlikely to drive ULIs.

We next tested if transcription cofactors may be required for ULIs. BRD4 has been implicated in chromatin organization (Rosencrance et al. 2020; Linares-Saldana et al. 2021), and both Mediator and BRD4 have been shown to be unevenly distributed in the nucleus and to be enriched in so-called transcriptional condensates (Cho et al. 2018; Sabari et al. 2018). However, our analysis shows that neither BRD4 degradation (Linares-Saldana et al. 2021) nor Mediator disruption (El Khattabi et al. 2019) affects ULIs (Fig. 5B,C). YY1 was implicated as a regulator of enhancer–promoter contacts (Weintraub et al. 2017), but a later report showed that degradation of YY1 had no effect on these (Hsieh et al. 2022). Using data from the latter study, we also found no effect of YY1 on ULIs (Supplemental Fig. S7B). Treatment with the EP300 catalytic inhibitor A-485 (Pelham-Webb et al. 2021) leads to loss of H3K27ac but has no effect on ULIs (Fig. 5D).

We considered whether ULIs may be driven by association with nuclear speckles, as this is strongly correlated with gene activity (Chen et al. 2018). Knockdown of the splicing component *Srrm2* leads to a partial disruption of speckles (Hu et al. 2019b), but this did not grossly affect ULIs (Supplemental Fig. S7C). Nuclear speckle association is primarily seen in the A1 subcompartment (Chen et al. 2018). We split active regions (H3K27ac peaks) in GM12878 cells by A1 and A2 (Rao et al. 2014) association and saw a higher level of ULI enrichment in A2 (Supplemental Fig. S7D). These data suggest that speckles cannot fully explain ULIs, although we cannot exclude that they may have some contribution.

Active regulatory elements, including CGIs, tend to be preferentially located toward the nuclear interior (Boyle et al. 2001; Beck et al. 2018). To test whether ULIs are a reflection of a more central nuclear position of these regions, we used GPSeq data from HAP1 cells, which measures radial positioning genome-wide (Girelli et al. 2020). We divided GPSeq regions into three bins (1–3) from the periphery to the center of the nucleus. As expected, CGIs lacking H3K27me3 in HAP1 cells were enriched in the most central GPSeq bin (Supplemental Fig. S7E). Analysis of pileups between regions in the three bins revealed no trend toward more interactions between centrally occupying regions, in *cis* or in *trans* (Supplemental Fig. S7F). However, this analysis normalizes the signal to the corners of the pileup to see enrichment compared with surrounding chromatin. When excluding this step and looking simply at average O/E contact frequencies in the different bins, interactions are highest between regions in the most central bin at the largest genomic distances (25–100 Mb) and in *trans* (Supplemental Fig. S7G). This likely reflects the higher level of chromosome intermingling in the center of the nucleus (Kalhor et al. 2011). Our analyses indicate that (1) interactions between CGIs happen more often in the center of the nucleus because they tend to be localized there, (2) focal enrichment in contact frequencies between CGIs over the surrounding chromatin is similar regardless of nuclear radial positioning measured by GPSeq, and (3) *trans* or very distal *cis* interactions are higher between regions in more centrally occupying regions. We therefore conclude that radial positioning contributes to, but does not fully explain, ULIs.

As levels of transcription are correlated to ULIs, we investigated whether loss of transcription would disrupt ULIs. Data from mESCs treated with flavopiridol or triptolide (Hsieh et al. 2020), which block transcription elongation and initiation, respectively, for 45 min did not show a loss of ULIs (Fig. 5E). Neither did degradation of RPB1, the largest subunit of Pol II, for 6 h (Fig. 5F; Jiang et al. 2020). ULIs are lost in mitosis and regained before cells enter G_1_ (Supplemental Fig. S5A). When analyzing data from cells that were synchronized in mitosis and then released into G_1_ with or without RPB1, we saw ULIs in both conditions (Fig. 5F). These results for long-range interactions are similar to the conclusion that inhibition of transcription has a modest global impact on shorter-range promoter–promoter and enhancer–promoter contacts (Hsieh et al. 2020; Jiang et al. 2020; Goel et al. 2023), although prolonged Pol II depletion can lead to the reduction of some enhancer–promoter contacts owing to changes in cohesin binding (Zhang et al. 2023).

### Simulations of multivalent binding are consistent with observed long-range interactions

**Figure 6.**
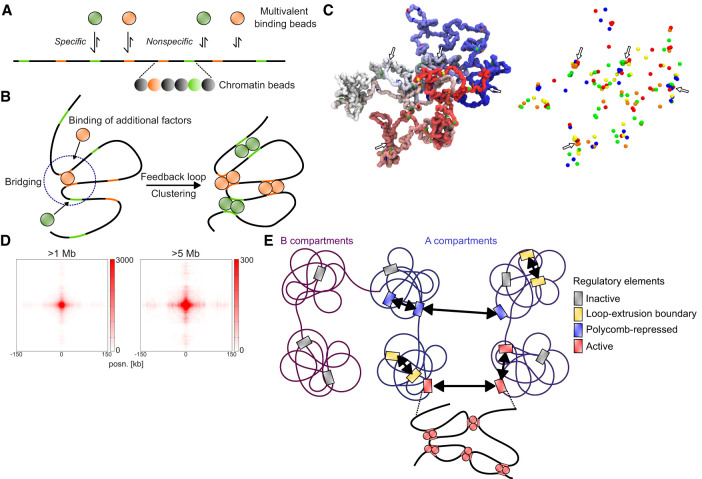
Simulations of chromatin and multivalent binding proteins. (*A*) Schematic depiction of the model with different multivalent binding beads (*top*) that bind specifically to colored binding sites and nonspecifically to other chromatin beads (*bottom*). (*B*) Schematic illustration of bridging-induced clustering. Binding of a multivalent factor leads to bridging and increased concentration of chromatin and binding sites in the vicinity of the bound site. This leads to increased likelihood for more factors to bind, causing a feedback loop and clustering. (*C*) Snapshot of a configuration in the model. *Left* side represents chromatin (polymer colored from red to blue), with specific binding sites represented by differently colored beads. *Right* side represents only the specific binding sites. Instances of clustering are highlighted with arrows. (*D*) Cumulative contacts in virtual Hi-C between binding sites in the simulations at distances >1 Mb (*left*) and >5 Mb (*right*). (*E*) Proposed model for interactions between CREs. Inactive genes in A or B compartments do not interact. In the A compartment, CREs overlapping loop extrusion boundaries interact via cohesin at short range, and polycomb-repressed CREs interact at short and long range. Active regulatory elements interact with each other at both short and long range, possibly through bridging-induced clustering

In an attempt to understand potential microscopic mechanisms underlying ULIs, we turned to simulations of chromatin. We considered a model in which binding and unbinding of multivalent factors to chromatin lead to “bridging-induced clustering,” without any affinity between the factors themselves (Fig. 6A; Brackley et al. 2016). This occurs because multivalent binding causes looping of chromatin and increased concentration of chromatin and binding sites near the already bound sites (Fig. 6B). This causes a feedback loop whereby more factors bind, further increasing the concentration of chromatin and binding sites. To test whether such a model would lead to increased interactions also between very distal binding sites, we modeled a chromatin fiber consisting of 5000 beads (3 kb per bead, representing 15 Mb of chromatin in total) using a bead-and-spring polymer model. In the fitting-free model, 172 of the beads represent randomly distributed binding sites for five different multivalent binding factors. Multivalent binding factors (200 in total, 40 per type) diffuse and bind with low affinity to all beads (nonspecific binding) and with high affinity to their corresponding binding sites.

We ran 180 simulations and detected clustering, as described previously (Fig. 6C). We then generated virtual Hi-C plots (Supplemental Fig. S8A) and analyzed total interactions between binding sites. We detected enrichment between binding sites at >1 Mb and >5 Mb distance, as well as a stripe emanating from the binding sites, similar to what is observed in Hi-C/Micro-C data (Fig. 6D). We considered what could lead to the stripe pattern seen in both the Hi-C/Micro-C data and our simulations. When we performed the simulations without the nonspecific affinity of binding factors to chromatin beads, retaining only specific binding, the central enrichment between sites was weaker, and the stripe pattern was almost completely gone (Supplemental Fig. S8B). This indicates that nonspecific binding is a strong contributor to the overall interactions and the stripe pattern.

Enriched interactions in the simulations occur between binding sites of both the same color (homotypic) and different colors (heterotypic) (Supplemental Fig. S8C). This is expected, as bridging-induced clustering exploits nonspecific interactions between all different binding factors and chromatin, so that mixed clusters form (see Fig. 6C). If we simulate the system with a single binding factor and, concomitantly, more separated binding sites, the enrichment and stripe pattern is weaker (Supplemental Fig. S8D). One prediction of the model is therefore that clustering depends on the distance to other binding sites and that isolated CREs would undergo less bridging-induced clustering than CREs with many nearby CREs. To test this, we took H3K27ac peaks in mESCs with different number of H3K27ac peaks within 100 kb, and sampled peaks to have similar enrichment for H3K27ac in the different groups (Supplemental Fig. S8E). We generated pileups for these regions with different numbers of “neighbor” sites at 1- to 100-Mb distances using Micro-C data. In agreement with the prediction, more isolated peaks had lower enrichment of interactions than did peaks with more neighboring sites (Supplemental Fig. S8F).

We next determined how often two distal regions in the model were in close proximity. We found that although interactions between distant binding sites for factors are enriched, occurrences of colocalization (<200 nm) between simulated FISH probes situated around such sites are rare as in our experimental data (1% and 4% for distances of 5.9 and 1.7 Mb, respectively) (Supplemental Fig. S8G,H).

In summary, our chromatin simulations with multivalent binding factors are consistent with several aspect of ULIs. These include enrichment for long-range (>1 Mb) interactions represented by rare colocalization events, a stripe pattern, no requirement for homotypic binding, and a correlation with the number of nearby active sites.

## Discussion

Overall, our results indicate that active CREs interact across large genomic distances, and the level of these interactions scale with activity: levels of transcription or H3K27 acetylation. ULIs are dynamic, changing between cell types and upon stimulation on the timescale of a few hours. There must therefore be some driving event leading to ULIs, which also coincides with activation of the genomic loci involved. Our simulations indicate that this may involve multivalent binding factors, which lead to bridging-induced clustering.

Although bridging-induced clustering requires no protein–protein interactions, it is heavily dependent on multivalency in binding. Whether there are single multivalent proteins that could be responsible for such bridging at active CREs is unclear, and we have not been able to identify any individual proteins whose perturbation disrupts ULIs, including RNA Pol II and several other cofactors involved in transcriptional regulation, including BRD4, Mediator, and EP300 activity. A more likely alternative is that effective multivalency could be achieved through different components in a complex that bind together. The model could also be compatible with a scenario in which protein–protein interactions lead to clustering of molecules, for example, through liquid–liquid phase separation, which can then bind to multiple sites.

Many other studies have observed CRE–CRE interactions at distances >1 Mb, through both conformation capture and imaging experiments (e.g., Osborne et al. 2004; Schoenfelder et al. 2010, 2015; Brackley et al. 2016; Bonev et al. 2017; Ogiyama et al. 2018). Those long-range interactions that cannot be explained by polycomb likely reflect the same aspect of genome organization we describe here, namely, the tendency of active regions to interact. CGI-containing genes have been shown to interact more than non-CGI genes within and across chromosomes (Beck et al. 2018). Interactions between promoter CGIs and nonpromoter (orphan) CGIs have also been implicated in driving transcriptional activation, although at much shorter genomic distances (Pachano et al. 2021). Although ULIs are particularly strong at CGIs, we see interactions between CGIs and other non-CGI active regions, which is consistent with the lack of specificity observed in our computational modeling with different binding factors.

Two recent studies describe small-scale compartments uncovered at very high sequencing depth either using whole-genome Hi-C or using Micro-C with capture of individual TADs (Goel et al. 2023; Harris et al. 2023). These results and ours may be different manifestations of the same underlying phenomenon. In line with this, they all occur at active enhancers and genes, and both microcompartments and ULIs appear unaffected by loss of loop extrusion or transcription. Although we propose the ultralong range as a defining feature of active CRE–CRE interactions, we believe the same process leads to interactions at shorter distances, such as the microcompartments within and between TADs (Goel et al. 2023). Indeed, our initial screen showed enrichment for factors associated with active regions both at the short range and long range, with a correlation between the two, and the proposed bridging-induced clustering model also leads to interactions at both short and long distances.

DNA FISH of CRE pairs several megabases apart showed only two instances of colocalization (<200 nm) of spots and marginally smaller median distances compared with CRE–control probes in the same A compartment. We note that contacts from chromosome capture experiments do not always reflect a high degree of colocalization in DNA FISH (Acuña et al. 2023). Rare colocalization was also detected in the simulations that gave rise to average interactions, consistent with ULIs. Therefore, ULIs should not be considered as stable interactions between individual regions, but rather an increase of already small probabilities of association between CREs compared with surrounding chromatin. It is the cumulative increase in these probabilities between many regions that gives rise to the average enrichment in Hi-C/Micro-C data.

Although all Hi-C/Micro-C protocols rely on formaldehyde cross-linking, we cannot think of a particular reason why active CREs would be more susceptible to enriched contacts as an artifact of this than other genomic regions, given that we find no evidence of correlation with accessibility or number of factors bound. Furthermore, our simulations provide a potential explanation for these observations that does not rely on any of the steps performed to generate contact frequencies experimentally.

We propose the following model for interactions between CREs across scales (Fig. 6E). At the ∼1 to 2 Mb scale, CREs are brought into proximity by the loop extrusion process, with focal interactions between loop extrusion boundaries such as CTCF sites. Polycomb-repressed regions are both locally compacted and interacting with each other across all genomic distances. Finally, active CREs, including promoters and enhancers, have enriched interactions with each other across all genomic distances, possibly driven by bridging-induced clustering. When two regions are in close enough proximity in the genome, these interactions could be frequent enough to have a functional impact. Furthermore, the cumulative interactions between all active CREs, including at large distances, are substantial and may have a functional effect. To test this, it would be necessary to be able to modulate contact frequencies between many regions without otherwise perturbing them. Possible avenues for exploration include targeting synthetic multivalent binding proteins to many sites in the genome to test the ability of multivalent binders to drive bridging-induced clustering and, if so, the potential impact of the gained interactions on transcription.

## Methods

### Hi-C data processing

Already processed Hi-C and Micro-C data were, when required, converted to cooler format (Abdennur and Mirny 2020) using hic2cool (Dekker et al. 2017) or *cooler cload* pairs (see Supplemental Table S3). Hi-C pairs from allele-phased SNPs (Han et al. 2020) were downloaded from the NCBI Gene Expression Omnibus (GEO; https://www.ncbi.nlm.nih.gov/geo/) under accession number GSE132898 and split into separate files by genotype annotation, followed by conversion to cooler using *cooler cload pairs*. Other data sets (see Supplemental Table S4) were processed using the distiller-nf 0.3.3 (https://github.com/open2c/distiller-nf) pipeline with default settings, and coolers filtered for mapq ≥ 30 were used. ICE balancing (Imakaev et al. 2012) was performed using *cooler balance*, using “*‐‐cis_only*” or “*‐‐trans-only*” to generate *cis*/*trans* balanced weights. Replicate data sets were combined when available using *cooler merge*. Expected contact frequencies were generated using the cooltools 0.5.1 (Open 2C et al. 2022a) functions expected-*cis* or expected-*trans* with “*‐‐clr_weight_name*” set to the appropriate balancing (*cis*/*trans*/total). Hi-C data in Figure 3C were visualized using HiGlass in resgen.io (Kerpedjiev et al. 2018).

### Contact screen

ReMap2022 peaks were downloaded from https://remap2022.univ-amu.fr/ (Hammal et al. 2022). Cistrome DB “factor” data for mouse and human were downloaded in batch from http://cistrome.org/db (Mei et al. 2017). Cistrome DB data sets corresponding to perturbation data sets (e.g., siRNA, KO, treatments) or misannotated data were discarded (see Supplemental Table S5). ReMap2022 and Cistrome DB data for mouse or human were combined and overlapped with ENCODE CREs (peaks within 5 kb merged using BEDTools *merge*) (Quinlan and Hall 2010) overlapping DNase-seq peaks in mESCs or GM12878, respectively (see Supplemental Table S6). O/E contact frequencies at 10-kb resolution (balanced using all contacts) and their respective coordinates for DNase-accessible CREs were extracted using coolpup.py (Flyamer et al. 2020) with the settings “*‐‐store_stripes ‐‐flank 0 ‐‐expected expected_file ‐‐mindist 100000 ‐‐maxdist 10000000 ‐‐by_distance 100000 1000000 10000000*.” For each data set in the Cistrome DB and ReMap2022 data, the contact frequency scores for short range and long range were split between pairs overlapping the data set at both ends or at neither end. Data sets with fewer than 500 regions or with fewer than 50 overlapping or nonoverlapping regions were discarded. A Mann–Whitney *U* test (using *scipy.stats.mannwhitneyu* with default settings) was performed to compare the two distributions and the effect size (*F*) calculated using *F* = U/n_1_ × n_2_, where n_1_ and n_2_ are the number of observations for overlapping and nonoverlapping regions. Adjusted *P*-values were calculated by multiplying the Mann–Whitney *U P*-value by the number of tests performed. TFs and transcription cofactors were derived from the AnimalTFDB 3.0 (Hu et al. 2019a) database followed by additional manual annotation (see Supplemental Tables S1, S2). For Supplemental Figure S1, the *F*-values were normalized to the mean *F* in each data set (e.g., *Rnf2* KO), and this normalized value was used to divide treated over untreated or KO over WT. For Figure 5A, the analysis was performed in the same way as described above, except peaks were combined for all ChIP-seq data sets of the same factor. Comparison was made between regions unbound by any TF to those bound on both sides by the factor or only one side but at least one other TF bound on the other side.

### Pileups

Pileups were generated with coolpup.py (https://github.com/open2c/coolpuppy, using v1.0.0 unless otherwise stated) (Flyamer et al. 2020). Most pileups are generated with the command “*coolpup.py cool_file peak_file ‐‐flank 100000 ‐‐mindist 100000 ‐‐maxdist 100000000 ‐‐by_distance 100000 1000000 10000000 25000000 100000000 ‐‐expected expected_file*.” Balancing (ICE normalization) was always performed on *cis* contacts, except for Supplemental Figure S6A, in which *trans* contacts were used, and Supplemental Figure S6B, in which all contacts were used. For all pileups, 5-kb resolution data were used, except for Figure 2C, in which 100-bp resolution was used. For Supplemental Figure S3E, mindist was set to 1,000,000, and clr_weight_name was altered based on if using total, *cis*, or no balancing, and “*‐‐nshifts 0*” “*‐‐nshifts 5*” *or* “*‐‐expected expected_file*” was used. Central pixel interactions and stripes in Figure 3, A and B, were generated using “*‐‐mindist 10000000 ‐‐maxdist 25000000 ‐‐store_stripes.*” For Supplemental Figure S4C, regions of interest were overlapped with TADs using *bioframe.overlap*. coolpup.py was run using “*groupby = [“TAD”]*,” where TAD represents a unique value for each TAD in order on the genome. The number of TADs separating the two regions were calculated using a custom modify_2Dintervals_func in coolpup.py. For Supplemental Figure S6C, regions near compartment edges were annotated as + or − stranded depending on if the nearest boundary was to the left or right, and pileups were generated with “*‐‐by_strand ‐‐mindist 1000000 ‐‐maxdist 100000000 ‐‐flip_negative_strand*” and combining −− with ++ using a custom modify_2Dintervals_func in coolpup.py. For figures comparing interactions between different sets of regions, for example, Figure 2G, the different sets were annotated as + or −, and pileups were generated with “*‐‐by_strand ‐‐flip_negative_strand*” and combining +− and −+ using a custom modify_2Dintervals_func in coolpup.py. For Supplemental Figure S8C, pileups were generated on peaks annotated by the number of neighbors and binned (see Peaks and Coverage Files section) using the settings “*‐‐expected expected_file ‐‐mindist 1000000 ‐‐maxdist 100000000 ‐‐groupby neighbor_bin1 neighbor_bin2 ‐‐ignore_group_order*” and using coolpup.py v1.1.0. Pileup heatmaps were generated using the coolpuppy function plotpup.py with the settings “*‐‐plot_ticks*” and appropriate values for “*‐‐cols*” and “*‐‐rows*.” “*‐‐norm_corners 10*” was used in all cases except for Supplemental Figures S3E, S7G, and S8F. Stripe plots in Figure 3B were plotted using plotpup.py with the settings “*‐‐stripe corner_stripe ‐‐plot_ticks ‐‐lineplot*.”

### Peaks and coverage files

Peaks (bed or narrowPeak) and coverage files (bigWig) used are listed in Supplemental Tables S6 and S7. Overlaps between peaks were generated using BEDTools *intersect* or *bioframe overlap* (Open2C et al. 2022b). Peaks were randomly sampled in some cases to get the same or similar number of peaks for comparisons. Distances to the closest peak were generated using BEDTools *closest*, and peaks were merged using BEDTools *merge*. For Supplemental Figure S3, A and B, and Figure 2F, ENCODE DNase-seq peaks or H3K27ac peaks not overlapping TSSs, CGIs, or RING1B were split into quartiles based on the signalValue column. CGIs and dinucleotide frequencies were derived from CAP-CGI data (Illingworth et al. 2010). TSSs were defined as the first TSS for all genes in refGene (O'Leary et al. 2016). For Supplemental Figure S4C, TAD boundaries were called at 250-kb resolution using *cooltools insulation*. Processed H3K27ac values from stimulated THP-1-derived macrophages (Reed et al. 2022) were split into quartiles based on the m0000_VST and m1440_VST columns, and regions with adjusted *P*-values < 0.05 and going from quartiles 1 or 2 to 4 (up-regulated) or 4 to 1 or 2 (down-regulated) were used. For Supplemental Figure S6C, compartments were called using *cooltools eigs-cis* at 50-kb resolution, and eigenvector E1 values above and below zero were called as A and B and adjacent bins in the same compartment merged. TSSs not overlapping RNF2 binding sites within 50 kb of compartment edges were compared to Q4 TSSs not overlapping RNF2 at least 100 kb from compartment edges. Zebrafish H3K27ac enrichment values were taken from Figure 2D source data from Yang et al. (2020) and brain/muscle enrichment defined as those with values greater than four in one and less than one in the other tissue. Processed *D. melanogaster* RNA-seq data from Loubiere et al. (2020) were split into quartiles based on the baseMean value. For GM12878, the top 20000 H3K27ac peaks (based on signalValue) not overlapping H3K27me3 from ENCODE were used and overlapped with ENCODE MYC peaks (Supplemental Fig. S7A) or subcompartments from Rao et al. (2014) (Supplemental Fig. S7D). GPSeq data at 100-kb resolution were downloaded from GitHub (https://github.com/ggirelli/GPSeq-source-data) (source data Fig. 2E), and values from two experiments were averaged and split into three bins, which were overlapped with CAP-CGI regions that do not overlap H3K27me3 in HAP1 (from ENCODE). For Supplemental Figure S8, E and F, the 50 nearest neighbors within ENCODE H3K27ac peaks were generated using BEDTools *closest* with the settings “*-d -k 50*.” Neighbors within 2.5 kb were discarded, and the number of neighbors within 100 kb were counted for each peak and divided into groups (zero, one to four, five to 10, 10 or more neighbors). The groups were sampled to generate equal numbers and similar distributions of H3K27ac based on the signalValue column using *nullranges* (Mu et al. 2023) in R with the settings “*covar* = ∼*signalValue, method = ‘stratified.’*” Peaks were converted between assemblies using UCSC liftOver (Hinrichs et al. 2006). Coverage heatmaps and lineplots were generated using deepTools *computeMatrix* with the settings “*reference-point ‐‐referencePoint center -a 5000 -b 5000*” and plotted using deepTools *plotHeatmap* or *plotProfile* (Ramírez et al. 2016).

### Expression quartiles and H3K27ac differential abundance analysis

4sU-seq data from mESCs and differentiating toward NPCs (Boyle et al. 2020) were aligned to the mm10 genome in paired-end mode using STAR 2.7.1a (Dobin et al. 2013) with the settings “*‐‐outFilterMultiMapNmax 1.*” H3K27ac ChIP-seq data from mESCs (Luo et al. 2020) and NPCs (Bonev et al. 2017) were aligned to the mm10 genome in single-end mode using STAR 2.7.1a with the settings “*‐‐outFilterMultiMapNmax 1 ‐‐alignMatesGapMax 2000 ‐‐alignIntronMax 1 ‐‐alignEndsType EndToEnd.*” Duplicate reads were discarded using Picard (http://broadinstitute.github.io/picard/), and reads not aligning to autosomes, X, or Y were removed using SAMtools 1.10 (Li et al. 2009). Read counts were generated using HOMER 4.10 (Heinz et al. 2010) annotatePeaks.pl with the settings “*-noadj -len 0 -size given*” in the first exon of every refGene gene (for 4sU-seq) or merged H3K27ac peaks within 5 kb from mESCs and NPCs (for H3K27ac ChIP-seq). TMM normalization was performed using edgeR (Robinson et al. 2010) in R 4.1.3 (R Core Team 2022). For 4sU-seq, the normalized values from both replicates were averaged, divided by the length of the exon, and split into quartiles. For H3K27ac, DE analysis using limma (Ritchie et al. 2015) was performed with contrast “∼*0 + Sample.*” Peaks with adjusted *P*-values < 0.05 were split into those higher in mESCs or NPCs.

### DNA FISH

mESCs were grown on 0.1% gelatin-coated slides in GMEM BHK-21 (Gibco 21710-025) supplemented with 15% fetal calf serum (Sigma-Aldrich F-7524), leukemia inhibitory factor (in-house), 2 mM L-glutamine (in-house), 1 mM sodium pyruvate (Sigma-Aldrich 58636), 1× nonessential amino acids (Sigma-Aldrich M7145), and 50 mM 2-β-mercaptoethanol (Gibco 31350-010). Cells were fixed with 4% paraformaldehyde for 10 min at room temperature, washed, permeabilized in PBS/0.5% Triton X-100, dried, and then stored at −80°C before hybridization. Slides were incubated in 100 μg/mL RNase A in 2× SSC for 1 h at 37°C, washed briefly in 2× SSC, passed through an alcohol series, and air-dried. Slides were incubated for 5 min at 70°C, denatured in 70% formamide/2× SSC (pH 7.5) for 40 min at 80°C, cooled in 70% ethanol for 2 min on ice, and dehydrated by immersion in 90% ethanol for 2 min and 100% ethanol for 2 min before air drying. One microgram of fosmid DNA was labeled by nick translation to incorporate green-dUTP (Enzo Lifesciences), Alexa fluor 594-dUTP (Invitrogen), or digoxigenin-11-dUTP (Roche). One hundred nanograms of each fosmid, 6 μL of Cot1 DNA per fosmid, and 5 μg of sonicated salmon sperm DNA were dried in a spin-vac and then reconstituted in 30 μL of hybridization mix. Probes were then denatured for 5 min at 80°C and reannealed for 15 min at 37°C. Fosmid probes were hybridized to slides under a sealed coverslip overnight at 37°C. Slides were washed the next day four times for 3 min in 2× SSC at 45°C and four times for 3 min in 0.1× SSC at 60°C, and the digoxigenin-labeled probe was detected with antidigoxigenin antibody (Roche 11333089001) and Alexa-Fluor 647 donkey antisheep antibody (Invitrogen A21448). Slides were stained with 4,6-diaminidino-2-phenylidole (DAPI) at 50 ng/mL, mounted in VectaShield (Vector Laboratories), and sealed with nail varnish. Slides were imaged on the SoRa spinning disk confocal microscope (Nikon CSU-W1 SoRa), and images were denoised and deconvolved using NIS deconvolution software (blind preset; Nikon). 3D images are shown in the figures as maximum intensity projections prepared using ImageJ (Schneider et al. 2012). The distances between the relevant spots were calculated using the Imaris v9.4 (Oxford Instruments) spot function. Statistical comparison was performed using a linear mixed-effect model using *nlme::lme* in R with “*fixed = distance*∼*category + chromosome, random* = ∼*1|replicate*,” where category refers to CRE–CRE or CRE–Control (*P* = 0.0325 for all data). For individual chromosome comparisons, only the values from those DNA FISH experiments were included and *lme* run with “*fixed* = distance∼category, *random* = ∼*1|replicate*” (*P* = 0.7777, *P* = 0.0686, *P* = 0.0431 for Chr 1, Chr 9, and Chr X, respectively). Probe coordinates are found in Supplemental Table S8.

### Chromatin simulations

The general outline of the model is similar to that of Brackley et al. (2021). For a detailed description of the model, refer to the Supplemental Methods. Example code is provided at GitHub (https://github.com/efriman/Friman_etal_ULI). Briefly, LAMMPS (Thompson et al. 2022) was used to perform molecular dynamics simulations of a beads-and-spring polymer representing chromatin with certain beads in the polymer corresponding to high-affinity sites for binding factors. Each bead in the chromatin polymer represents ∼3 kb or a physical size of ∼30 nm. Additional beads representing multivalent binding factors were modeled with different affinities to specific chromatin beads and all chromatin beads. Binding beads undergo both binding and unbinding events. Simulations were initialized with the chromatin fiber as a random walk and binding proteins randomly distributed. Virtual Hi-C, pileups, and FISH were generated from configurations in the second half of the simulated trajectories. For virtual Hi-C, a contact was considered if two regions of the chromatin fiber were within 90 nm and binned at 21-kb resolution (seven chromatin beads). For virtual pileups, the contact distance was 54 nm, and binning resolution was 3 kb (one chromatin bead). Contacts in the pileups are cumulative, and the values represent counts (i.e., number of contacts). Virtual FISH distributions were generated based on the center of mass distances between 51-kb regions surrounding the two sites.

### Software availability

The code used in the analysis is available at GitHub (https://github.com/efriman/Friman_etal_ULI) and as Supplemental Code. Custom analysis was performed in Python 3.8.12 (van Rossum and Drake 1995) in Jupyter notebooks (Kluyver et al. 2016) using pandas (McKinney 2010), numpy (Harris et al. 2020), and SciPy (Virtanen et al. 2020) for data analysis and Matplotlib (Hunter 2007) and seaborn (Waskom 2021) for plotting.

## Supplementary Material

Supplement 1

Supplement 2

Supplement 3

Supplement 4

Supplement 5

Supplement 6

Supplement 7

Supplement 8

Supplement 9

Supplement 10

Supplement 11
