# Supplementary material for: Ultra-long-range interactions between active regulatory elements
Source: Genome Res. 2023 Aug;33(8):1269–83. doi: 10.1101/gr.277567.122 (PMC10547262; doi:10.1101/gr.277567.122)
Supplement: Supplement 9 [file Supplemental_Methods.docx]

**Chromatin modelling**

For the polymer physics modelling, coarse-grained molecular dynamics simulations were performed, in which collections of molecules are represented by beads, interacting with phenomenological force fields. More specifically, a chromatin fibre was modelled as bead-and-spring polymers, while multivalent chromatin-binding (and bridging) proteins representing, for instance, complexes of transcription factors and polymerases, were represented by additional individual beads. We used the multi-purpose molecular dynamics package LAMMPS (Large-scale Atomic/Molecular Massively Parallel Simulator; Plimpton, 1995) to perform our simulations. We will now describe the potentials underlying the force fields used in the simulations presented in the text. Note that multiple colour and single colour model refer to model with 5 types and 1 type of chromatin-binding proteins respectively.

*The chromatin fibre*

A chromatin fibre, corresponding to a 15 Mbp (5 Mbp) chromatin region for the multiple colour (single colour) simulations was discretised as a set of monomers, each of size corresponding to 3 kbp, or to a physical size $\sigma\sim$30 nm (Brackley et al., 2021). There were different class of chromatin beads, as follows. A first class is weakly binding to all types of multivalent proteins, to model non-specific binding, which is an important ingredient in our force field. A second class binds specifically to multivalent proteins of one type. Below we specify when the potentials used depend on chromatin bead type. When nothing is specified, the potential is applied regardless of bead type.

Any two monomers (*i* and *j*) in the chromatin fibre interact purely repulsively, via a Weeks-Chandler-Anderson (WCA) potential, given by

$U_{ij}^{\text{WCA}}\left( r_{ij} \right)=\left\{ \begin{aligned} 4k_{B}T\left[ \left( \frac{\sigma}{r_{ij}} \right)^{12}-\left( \frac{\sigma}{r_{ij}} \right)^{6}+\frac{1}{4} \right] \mathrm{if}r_{ij}<2^{1/6}\sigma\\ 0 otherwise, \end{aligned} \right.$ (S1)

where $r_{ij}$ is the separation of beads *i* and *j*. There is also a finite extensible non-linear elastic (FENE) spring acting between consecutive beads in the chain to enforce chain connectivity. This is given by

$U_{ij}^{FENE}\left( r_{ij} \right)=-\frac{K_{f}R_{0}^{2}}{2}\ln\left[ 1-\left( \frac{r_{ij}}{R_{0}} \right)^{2} \right] ,$ (S2)

where *i* and *j* are neighbouring beads, $R_{0}=1.6\sigma$ is the maximum separation between the beads, and $K_{f}=30{k_{B}T}/{\sigma^{2}}$ is the spring constant. Additionally, a triplet of neighbouring beads in the chromatin fibre interact via a Kratky-Porod term to model the stiffness of the chromatin fibre, which explicitly reads as follows,

$U_{ij}^{\text{KP}}= \frac{k_{B}Tl_{p}}{\sigma}\left[ 1-\cos\theta_{ij} \right] ,$ (S3)

where *i* and *j* are neighbouring beads, while $\theta_{ij}$ denotes the angle between the vector connecting beads *i* and *j = i+1* and the vector connecting beads *j* and *j+1*. The quantity $l_{p}$ is related to the persistent length of the chain. We set it to $3\sigma$ in our simulation, which corresponds to a relatively flexible chain.

*Multivalent chromatin-binding proteins*

An important component of the model is constituted by multivalent chromatin-binding proteins, or bridges, which can bind to chromatin at multiple places. Chromatin bridges were modelled as spheres, again with size $\sigma$ for simplicity. We imagine these represent protein complexes, for instance of transcription factors and polymerases.

In our simulations, the interaction between a chromatin bead, *a*, and a multivalent chromatin-binding protein, *b*, was modelled via a truncated and shifted Lennard-Jones potential, given by

$U_{ab}^{\text{LJ}}\left( d_{ab} \right)=\left\{ \begin{aligned} \frac{4\epsilon_{ab}}{\mathcal{N}}\left[ \left( \frac{\sigma}{d_{ab}} \right)^{12}-\left( \frac{\sigma}{d_{ab}} \right)^{6}-\left( \frac{\sigma}{r_{c}} \right)^{12}+\left( \frac{\sigma}{r_{c}} \right)^{6} \right] \mathrm{if}d_{ab}<r_{c} \\ 0 otherwise, \end{aligned} \right.$ (S4)

where $d_{ab}$ denotes the distance between the centres of the chromatin bead and protein, $r_{c}$ is a cut-off parameter, and $\mathcal{N}$ is a normalisation constant which ensures the depth of the potential reaches $-\epsilon_{ab}$ at the minimum point. The exact values of $\epsilon_{ab}$ chosen are discussed below (see *Parameter values*). Chromatin-binding proteins interacted with each other only sterically (via the WCA potential).

Chromatin-binding proteins could switch back and forward between a binding and a non-binding state with a rate $k_{\text{sw}}$.This feature mimics post-translational modifications on protein complexes and accounts for the dynamical turnover of constituents within nuclear protein clusters (Brackley et al., 2017). When proteins were non-binding, they interacted with chromatin beads via the WCA potential.

*Parameter values*

Here we provide the remaining list of parameter values used in simulations.

Regarding chromatin-protein interactions, we used an interaction range $r_{c}=1.8 \sigma$ in all cases, with a value of $\epsilon_{ab}=2.7 k_{B}T$ for non-specific interactions, and a value of $\epsilon_{ab}=7.1 k_{B}T$ for specific interactions, as in (Brackley et al., 2021).

Multivalent chromatin-binding proteins could switch at a rate $k_{sw}=0.001 \tau_{B}^{-1}$ between active (binding) and inactive (non-binding), where the Brownian time $\tau_{B}=\sigma^{2}/D$ , with $D$ the diffusion coefficient of the beads. The Brownian time gives an order-of-estimate measure of the time it takes for a bead to diffuse across its own diameter, and it is this timescale which one can use to determine the mapping of simulation time to real time where required. For the multiple colour (single colour model) there were 200 (40) proteins, on average 100 (20) active and the same time inactive at any time. For the multiple colour model, there were 40 proteins of each type (again, 20 on average active and 20 inactive).

We randomly scattered specific binding sites for proteins along the chromatin fibre as described previously in (Brackley et al., 2021) for the single colour case. There were 172 and 23 binding sites for multiple colour and single colour simulation respectively. These were generated by randomly setting a bead as specific binding site with probability 1/30 (multiple colour) or 1/40 (single colour). For the multiple colour simulation, we also selected randomly to which of the 5 proteins types each specific binding site was attracted to. Once the sequence of binding sites was chosen, it was kept constant for all simulations, so that different simulations have the same sequence of non-specific and specific binding beads, and can be aggregated for averaging.

The simulation domain was a cubic box with linear size equal to $100 \sigma$.

*Additional data analysis in simulations*

We performed multiple molecular simulations with the force field described above lasting 10^5^ Brownian times, and we analysed the second half of these simulations to remove effect of the initialisation. Simulations were initialised with the chromatin fibre as a random walk and chromatin binding proteins randomly distributed in the simulation domain.

To generate virtual Hi-C from simulations, we considered configurations from the second half of each simulated trajectory, to remove memory of the initial condition. We recorded a contact between any two beads if they were within a crosslinking threshold, equal to $3 \sigma$ (similar results were obtained with different thresholds). Contact maps given in this work were then binned (bin size 21 kbp).

To create virtual pileups from simulations, we considered any pair of specific protein binding sites, and cumulated contact data for the corresponding pixel and neighbouring ones (150 kbp each way, using a crosslinking threshold of $1.8 \sigma$ in this case), thereby replicating in silico the corresponding procedure used for experiments. For multiple colour simulations, we also separately considered data corresponding to: (i) pileups surrounding pairs of any protein binding sites, (ii) pileups surrounding pairs of binding sites for proteins of the same type (homotypic pileups); (iii) pileups surrounding pairs of binding sites for proteins of different types (heterotypic pileups).

To generate distributions for virtual FISH from simulations, we considered pair of beads corresponding to specific binding sites, computed the centre of mass of a 51 kbp region surrounding them (corresponding to a FISH probe), and computed the distance between the two centres of mass. The virtual FISH distribution is the distribution of such centre of mass distances.

**References for Modelling SI section:**

Brackley, C. A., Gilbert, N., Michieletto, D., Papantonis, A., Pereira, M. C. F., Cook, P. R., and Marenduzzo, D. Complex small-world regulatory networks emerge from the 3D organization of the human genome. *Nature Commun.* 12, 5756 (2021).

Plimpton, S. Fast Parallel Algorithms for Short-Range Molecular-Dynamics. *Journal of Computational Physics* 117, 1–19 (1995).

Brackley, C.A., Liebchen, B., Michieletto, D., Mouvet, F., Cook, P.R. and Marenduzzo, D., 2017. Ephemeral protein binding to DNA shapes stable nuclear bodies and chromatin domains. *Biophysical journal*, *112*(6), pp.1085-1093.
