# Supplementary material for: Ultra-long-range interactions between active regulatory elements
Source: Genome Res. 2023 Aug;33(8):1269–83. doi: 10.1101/gr.277567.122 (PMC10547262; doi:10.1101/gr.277567.122)
Supplement: Supplement 10 [file Supplemental_Figures.pdf]

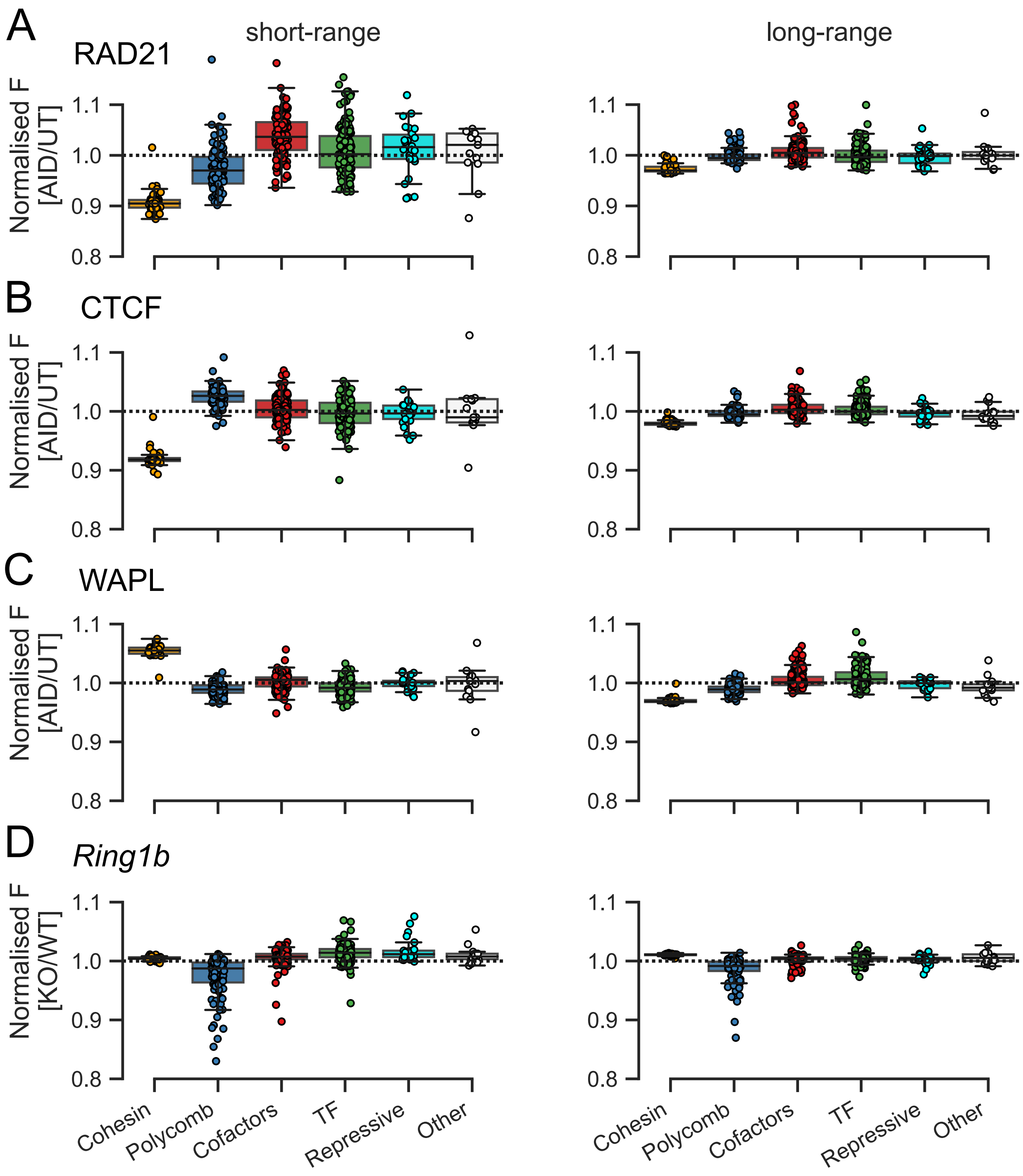

Supplemental figure S1

Supplemental figure S1. Effect of cohesin subunit degradation and *Ring1b* knockout on contact frequency screen enrichment values

(A-D) F-values for (left) short-range (0.1-1 Mb between pairs) and (right) long-range (1-10 Mb between pairs) enrichment were calculated for each Hi-C/micro-C dataset and the relative enrichment derived by dividing by the mean F for each dataset. This value was divided between treatment (AID = Auxin treated, or KO = knockout) and control (UT = Untreated, or WT) for **(A)** RAD21-AID, **(B)** CTCF-AID, **(C)** WAPL-AID, and **(D)** *Ring1b* KO.

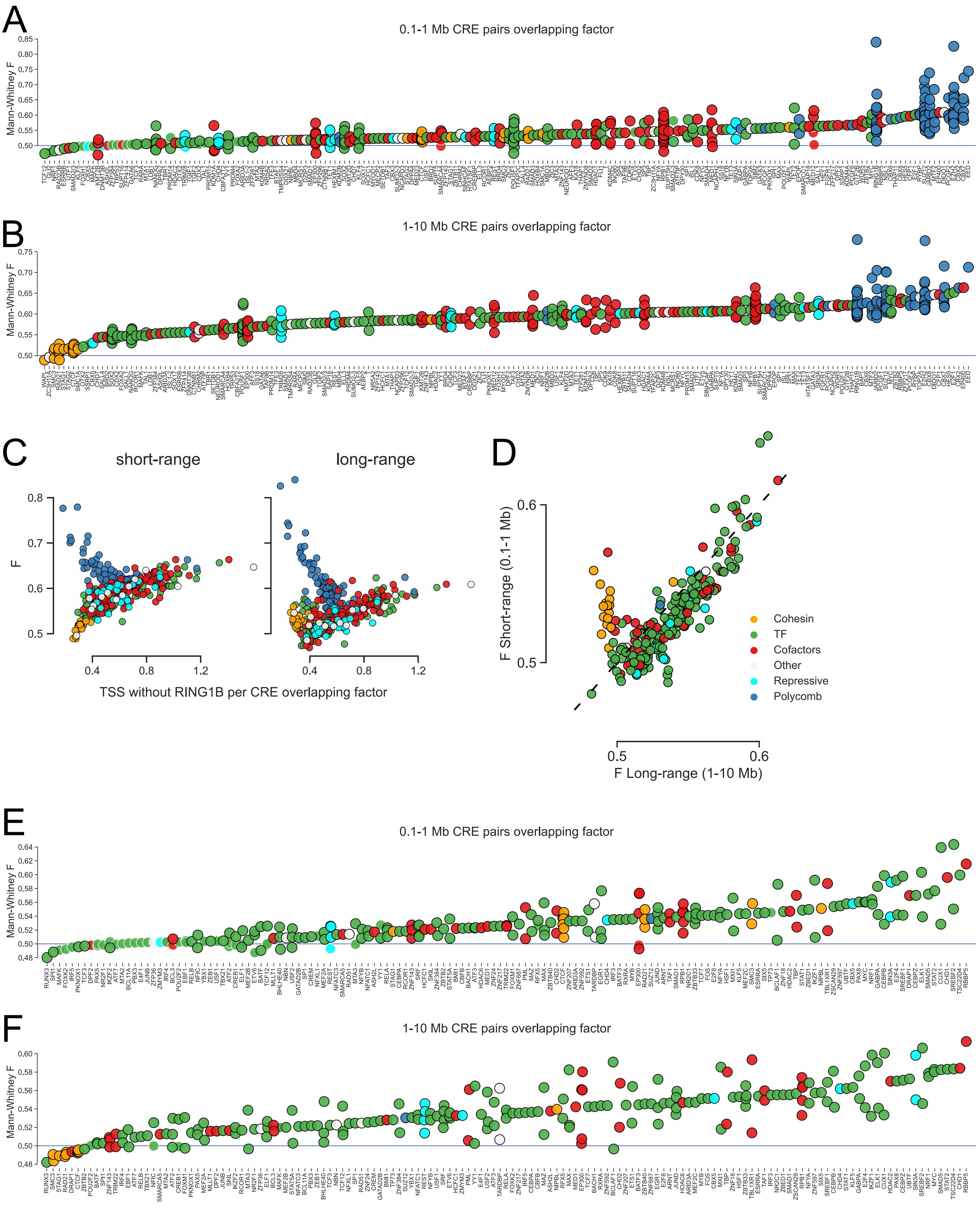

Supplemental figure S2

Supplemental figure S2. Contact frequency screen results in mESCs and human GM12878 lymphoblastoids

(A and B) F values for short-range **(A)** and long-range **(B)** enrichment in mESCs. Black outlined dots are significantly enriched (adjusted $p<0.05$), while non-outlined ones are not. **(C)** Correlation between the number of non-RING1B overlapping TSSs per CRE overlapping the factor and its enrichment (F) value at short (left) and long (right) range. **(D)** Effect sizes for factors with significantly enriched chromatin interactions compared to unbound CREs in the human GM12878 lymphoblastoid cell line. x and y axes show enrichment at short-range and long-range. Colours represent the group the factor belongs to. **(E and F)** Same as (A and B) but for GM12878.

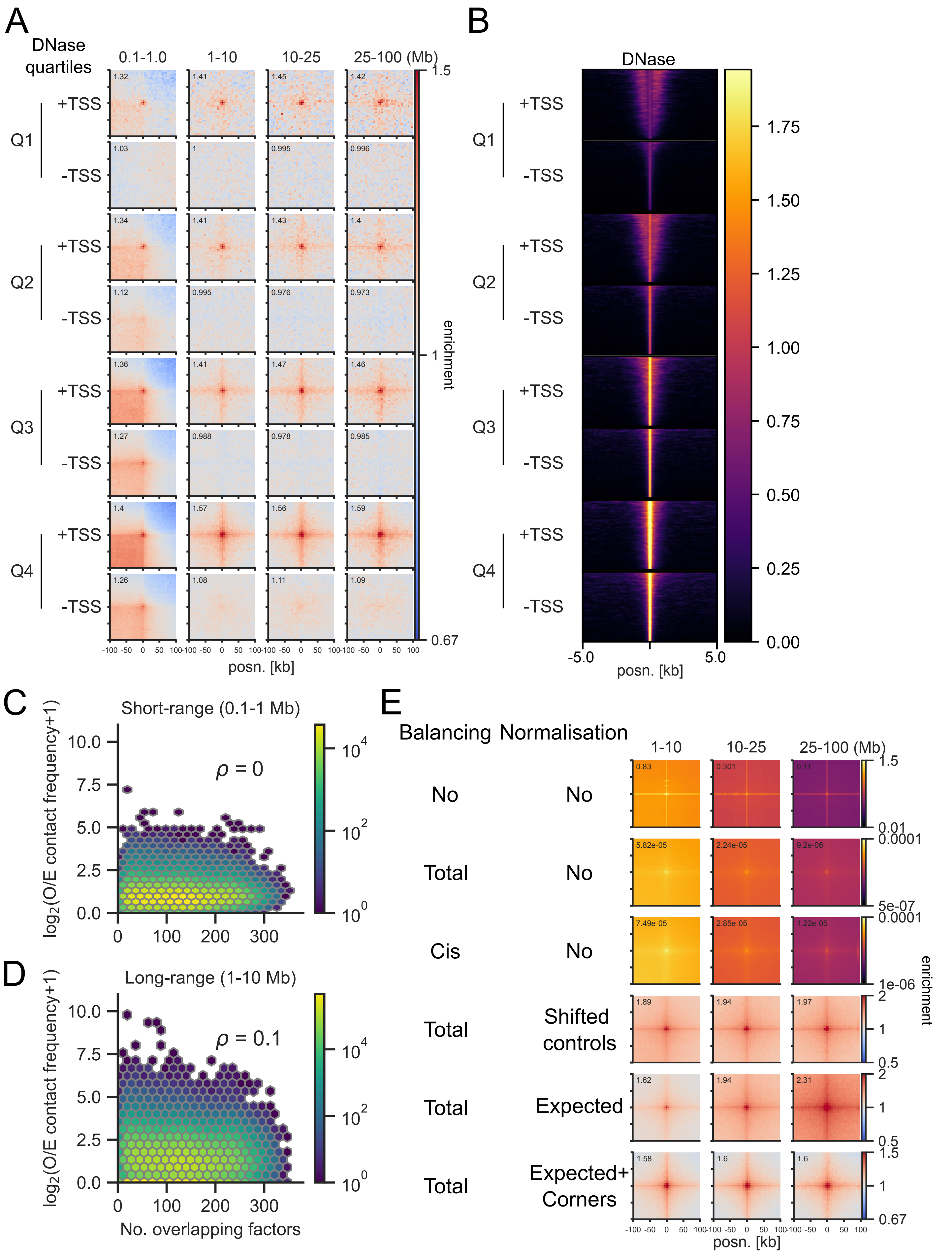

Supplemental figure S3

Supplemental figure S3. Exclusion of accessibility, binding, balancing, and normalisation as artifacts

(A) Pileup analysis of micro-C data from mESCs in quartiles split by DNase-seq signal and proximity to TSSs (<1kb or >5kb; 2705 peaks per group). **(B)** DNase-seq signal for regions used in (A). **(C and D)** Hexbin plots showing the correlation (ρ: Spearman's correlation coefficient) between the sum of the total number of factors overlapping the regions on both sides and contact frequencies at short-range **(C)** and long-range **(D)**. **(E)** Pileup analysis of 5898 CFP1 peaks not overlapping RING1B in micro-C data from mESCs using different balancing and normalisation parameters. Balancing refers to ICE normalisation, based on total or only cis contacts. Normalisation is performed using either 5 randomly shifted regions for each peak or based on calculated expected values. Corner normalisation refers to normalising the signal of each pixel by the average of the signal in the 10x10 corner pixels in the upper left and lower right corners.

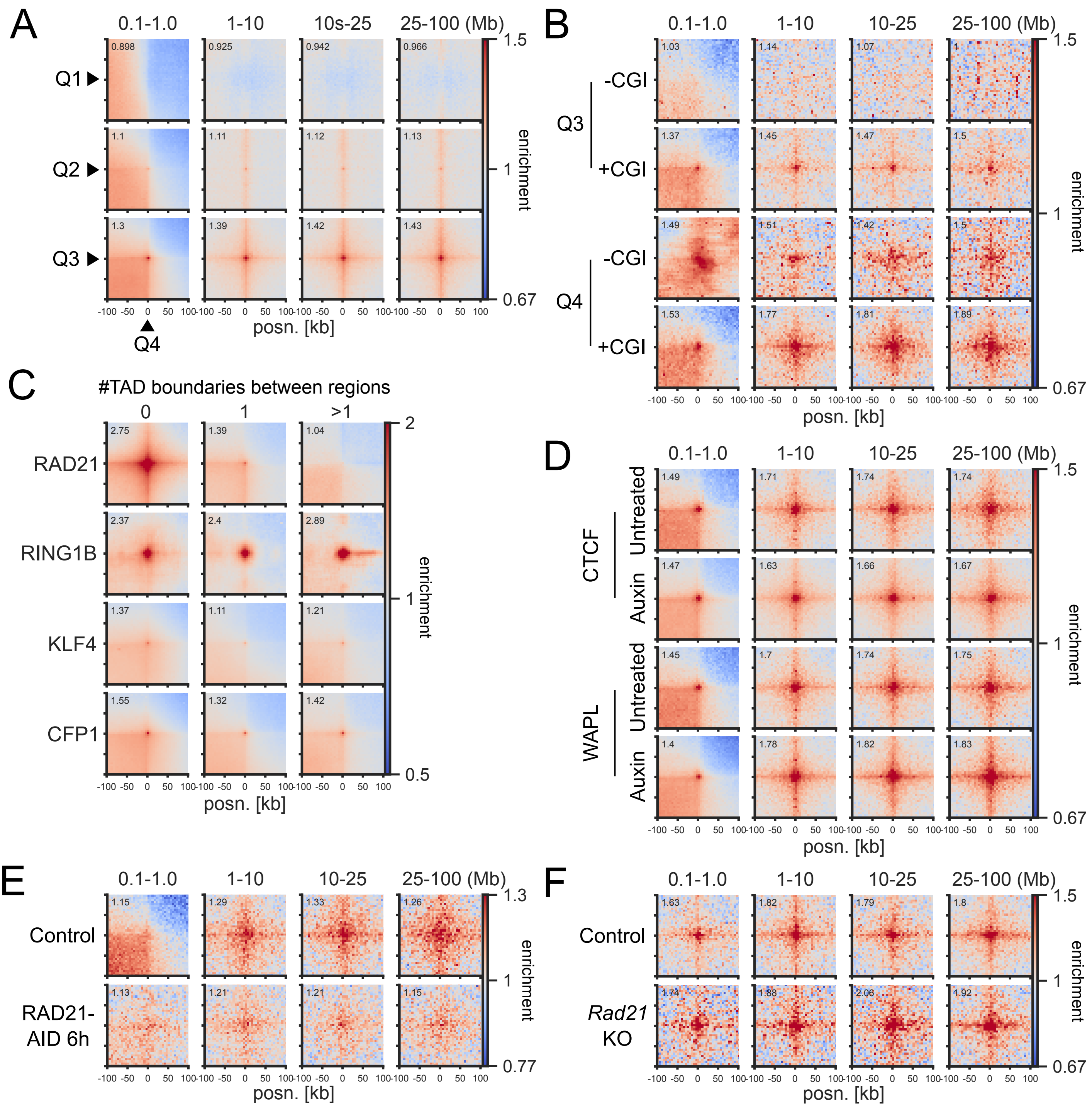

Supplemental figure S4

Supplemental figure S4. ULIs between TSSs, within/between TADs, effect of CTCF and WAPL degradation, and effect of RAD21 long-term depletion or knockout

(A) Pileup analysis between different non-RING1B bound TSS quartiles in micro-C data from mESCs. **(B)** Pileup analysis of the top two non-RING1B bound TSS quartiles based on expression and split by overlap with CGIs (732 peaks per group) in micro-C data from mESCs. **(C)** Pileup analysis for 5000 regions for each of the indicated hits from Fig. 1C for interaction pairs at 0.1-1 Mb genomic separation in micro-C data from mESCs, separated by number of TAD boundaries separating the regions. For CFP1 and KLF4, peaks overlapping RING1B binding sites were excluded. **(D)** Pileup analysis between CGI Q4 regions in micro-C data from CTCF-AID and WAPL-AID mESCs. **(E)** Pileup analysis between CGI Q4 regions in Hi-C data from RAD21-AID mESCs. **(F)** Pileup analysis between CGI Q4 regions in Hi-C data from WT and Rad21 KO thymocytes.

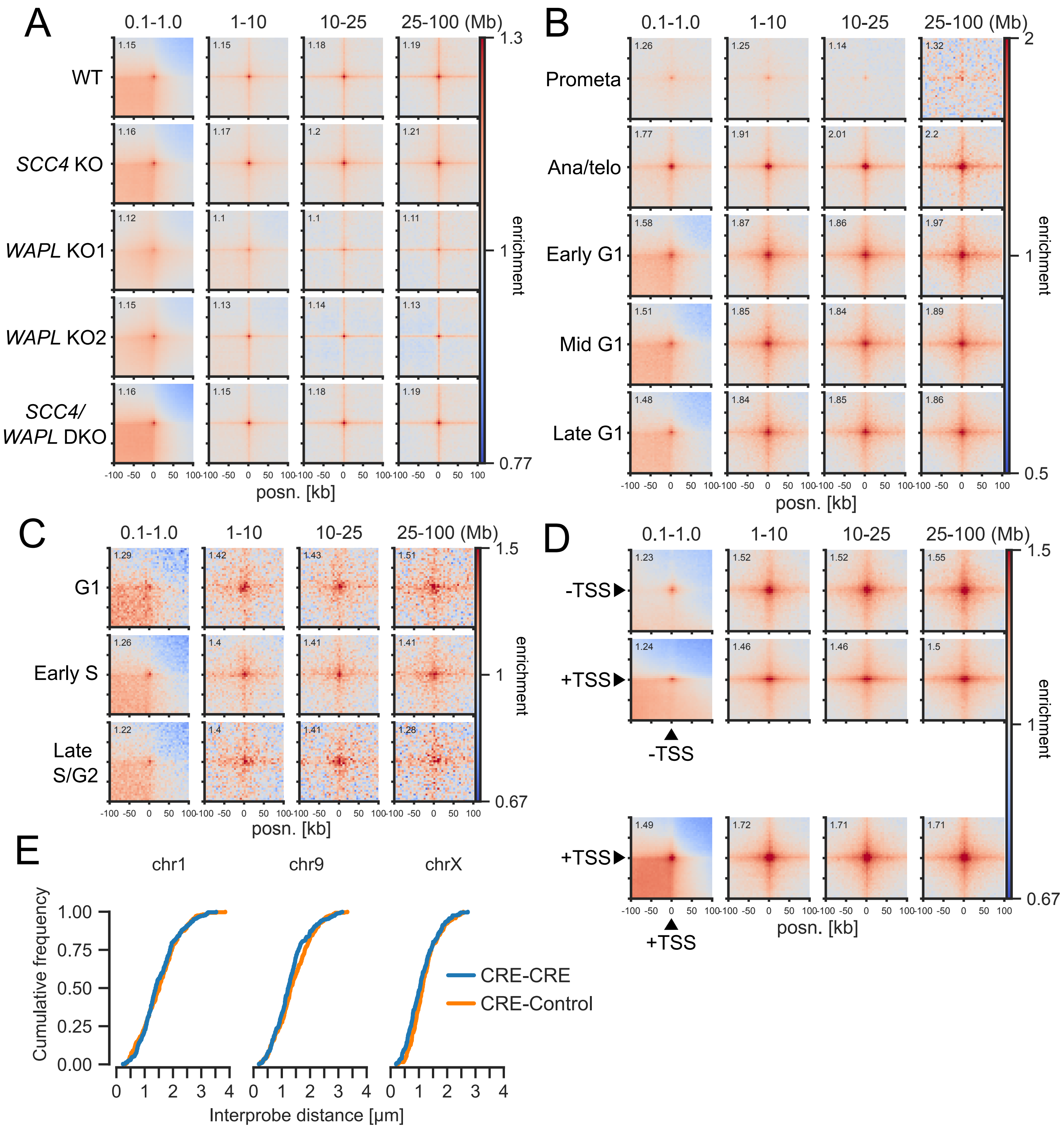

Supplemental figure S5

Supplemental figure S5. Cohesin disruption in HAP1, cell cycle phase, TSS-non TSS interactions, DNA FISH distributions

(A) Pileup analysis between high CpG density CGI (Q4) regions not overlapping H3K27me3 (22’924 Peaks) in Hi-C data from HAP1 cells with or without *SCC4* and/or *WAPL*. **(B)** Pileup analysis between CGI Q4 regions in Hi-C data from cell cycle staged mouse erythroblasts. **(C)** Pileup analysis between CGI Q4 regions in merged single-cell Hi-C data from cell cycle stage-inferred mESCs. **(D)** Pileup analysis between Q4 H3K27ac regions (6268 peaks; -TSS) and Q4 TSSs in micro-C data from mESCs. **(E)** Empirical cumulative distribution frequency of interprobe distances measured by DNA FISH between distal CREs on chromosomes 1, 9, and X or a CRE and an equidistant control region in the same A compartment.

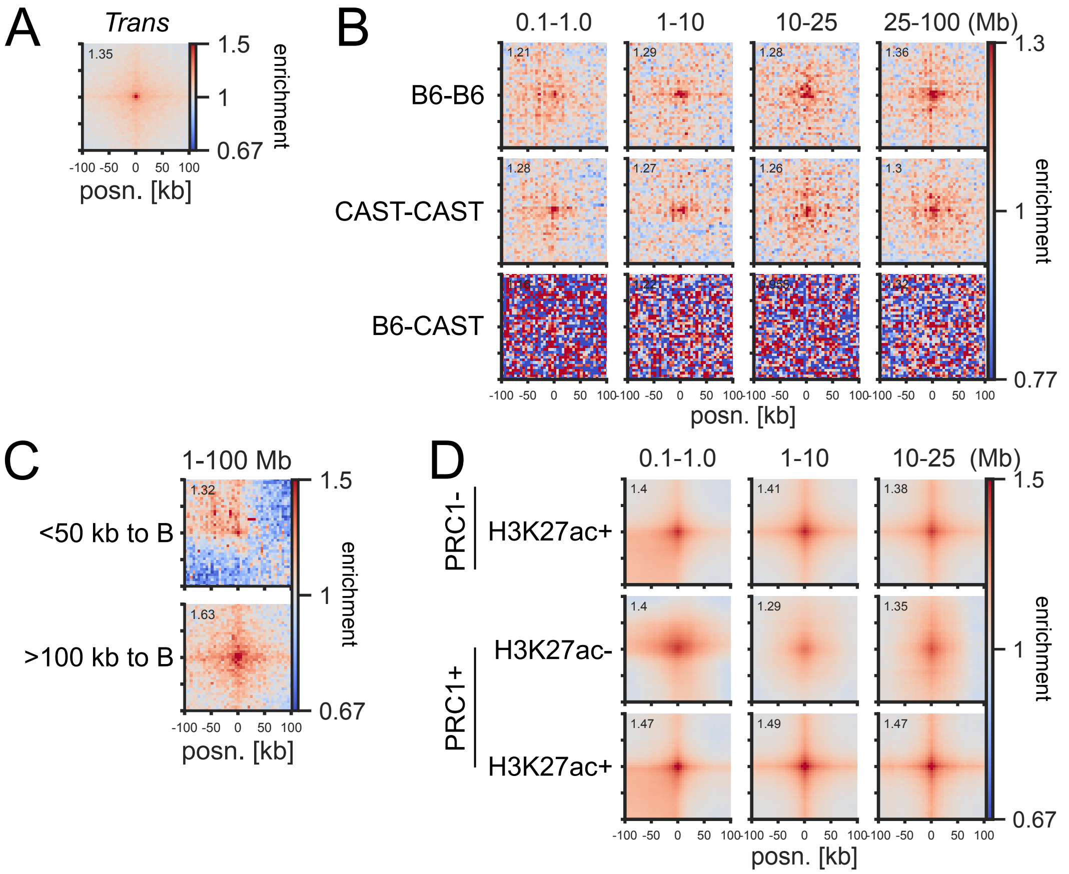

Supplemental figure S6

Supplemental figure S6. ***Trans*** **interactions, compartment switches, and interactions in** ***Drosophila melanogaster***

(A) Pileup analysis between CGI Q4 regions on different chromosomes (*trans)* in micro-C data from mESCs. **(B)** Pileup analysis for non-RING1B bound CGI regions (11’157 peaks) in Hi-C data from SNP-phased hybrid mouse CD4 T-cells. **(C)** Pileup analysis for non-RING1B bound TSSs close to B compartments (top; 1139 peaks) or Q4 TSSs far from B compartments (bottom; 1139 peaks) in micro-C data from mESCs. **(D)** Pileup analysis for regions with different combinations of H3K27ac and PRC1 (SUZ12 and PSC) regions (4722, 3187, and 3350 peaks) in Hi-C data from *D. melanogaster* eye-antennal imaginal discs.

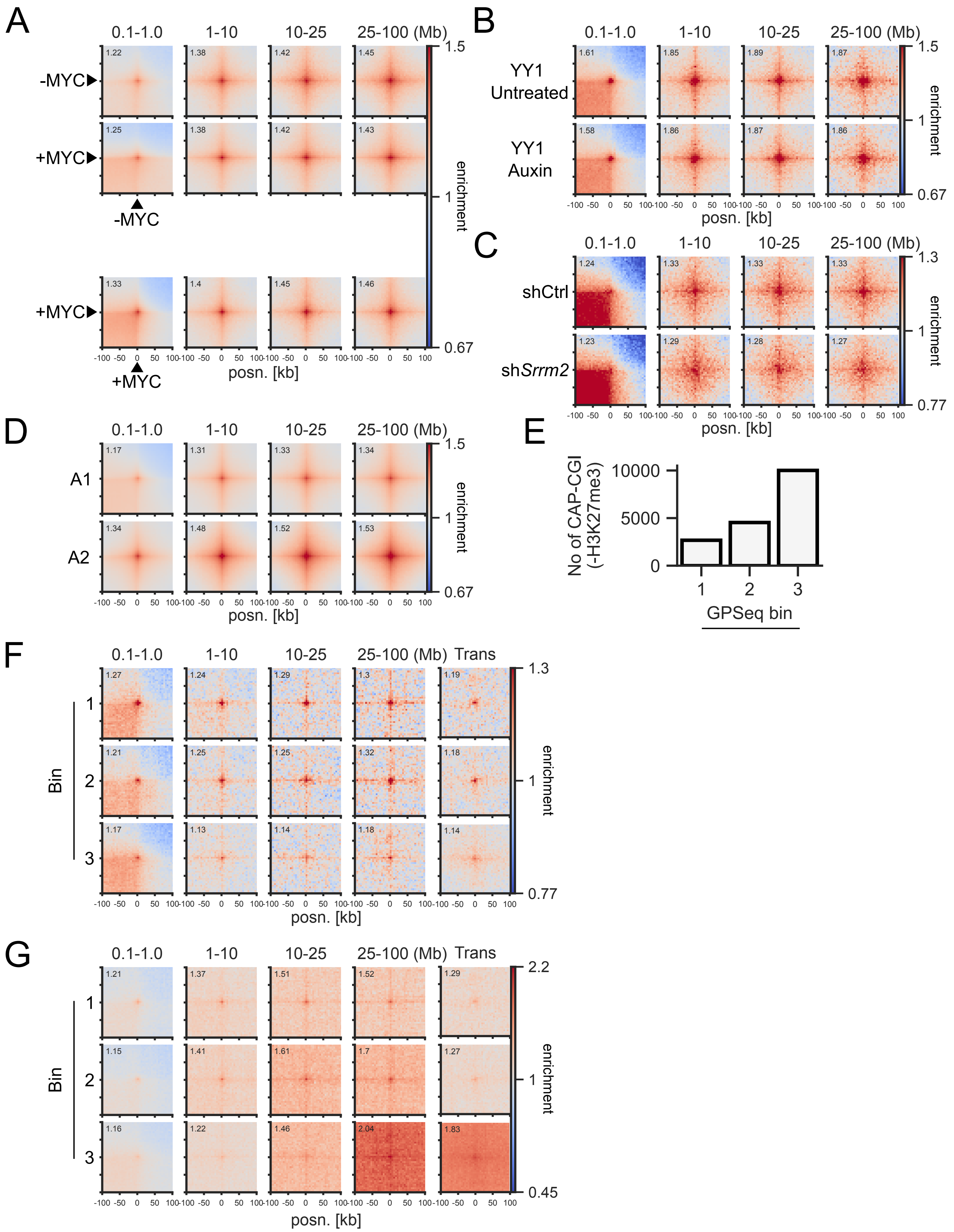

Supplemental figure S7

Supplemental figure S7. MYC binding, effect of YY1 and *Srrm2* perturbation, subcompartments, and correlation with radial positioning

(A) Pileup analysis between H3K27ac peaks with or without MYC (3301 peaks per group) in Hi-C data from GM12878. **(B)** Pileup analysis between CGI Q4 regions in micro-C data from YY1-AID mESCs. **(C)** Pileup analysis between CGI Q4 regions in Hi-C data from AML12 cells with control or *Srrm2* shRNA. **(D)** Pileup analysis of H3K27ac peaks overlapping the A1 (9445 peaks) or A2 (8082 peaks) subcompartments in Hi-C data from GM12878. **(E)** Number of CGIs not overlapping H3K27me3 in HAP1 overlapping three bins based on GPSeq signal, where higher means more central nuclear localisation. **(F)** Pileup analysis of CGIs overlapping different GPSeq bins (2670 peaks per group) in *cis* (left) and *trans* (right) in Hi-C from HAP1 cells. **(G)** Pileup analysis of CGIs overlapping different GPSeq bins in *cis* (left) and *trans* (right) in Hi-C from HAP1 cells without corner normalisation.

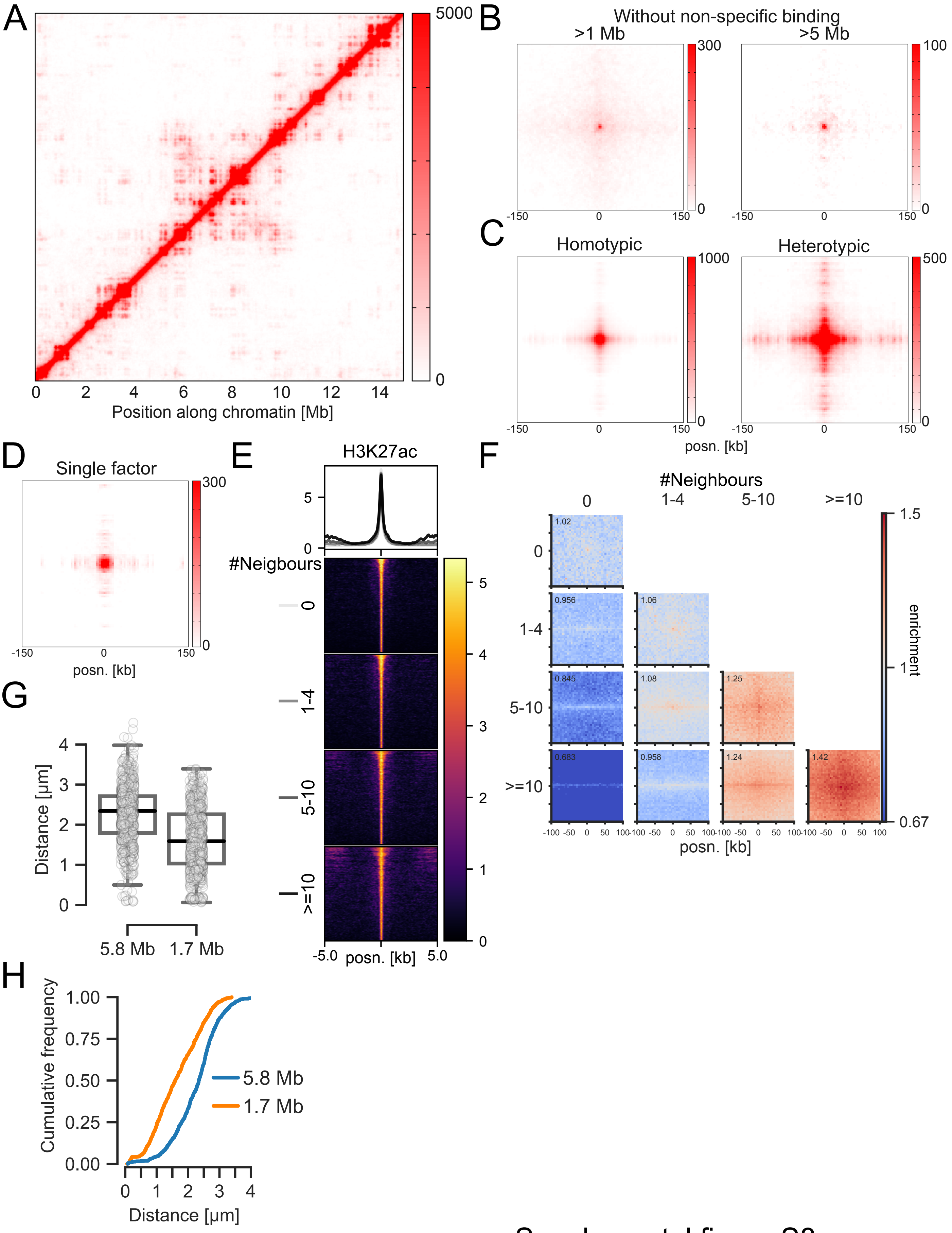

Supplemental figure S8

Supplemental figure S8. Results from simulations of chromatin and pileups between sites with different number of neighbours

(A) Virtual Hi-C map based on cumulative contacts in simulations. **(B)** Cumulative contacts in virtual Hi-C between binding sites in simulations excluding non-specific chromatin binding at distances >1Mb (left) and >5 Mb (right). **(C)** Cumulative contacts in virtual Hi-C between binding sites in the simulations, comparing homotypic (same binding sites) to heterotypic (different binding sites). **(D)** Cumulative contacts in virtual Hi-C between binding sites in a simulation with a single binding factor, where the binding sites are therefore more spread out. **(E)** H3K27ac signal at selected regions with different number of neighbouring H3K27ac peaks within 100 kb (1741 peaks per group). Top panel shows average and bottom shows individual regions in a heatmap. **(F)** Pileup analysis between H3K27ac regions in (E) in micro-C data from mESCs (without corner normalisation). **(G)** Distances between two binding sites at 5.8 and 1.7 Mb separation in individual time points across simulations. Boxplot lines denote maximum (excluding outliers), interquartile range (upper and lower bound of box), median (centre of box), and minimum values. **(H)** Empirical cumulative distribution frequency of distances in (G).
